# Sequence-Dependent Melting and Refolding Dynamics of RNA UNCG Tetraloops Using Temperature-Jump/Drop Infrared Spectroscopy

**DOI:** 10.1021/acs.jpcb.2c08709

**Published:** 2023-02-14

**Authors:** C. P. Howe, G. M. Greetham, B. Procacci, A. W. Parker, N. T. Hunt

**Affiliations:** † Department of Chemistry and York Biomedical Research Institute, University of York, Heslington, York YO10 5DD, U.K.; ‡ STFC Central Laser Facility, Research Complex at Harwell, Rutherford Appleton Laboratory, Harwell Science and Innovation Campus, Didcot OX11 0QX, Oxon, U.K.

## Abstract

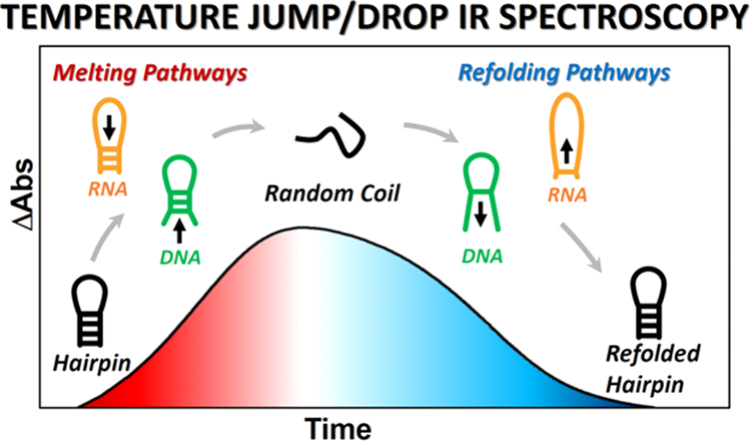

Time-resolved temperature-jump/drop infrared (IR) spectroscopy has been used to measure the impact of stem base sequence on the melting and refolding dynamics of ribonucleic acid (RNA) tetraloops. A series of three 12-nucleotide RNA hairpin sequences were studied, each featuring a UACG tetraloop motif and a double-stranded stem containing four base pairs. In each case, the stem comprised three GC pairs plus a single AU base pair inserted at the closing point of the loop (RNA_loop_), in the middle of the stem (RNA_mid_), or at the stem terminus (RNA_end_). Results from analogous DNA tetraloop (TACG) sequences were also obtained. Inclusion of AU or AT base pairs in the stem leads to faster melting of the stem-loop structure compared to a stem sequence featuring four GC base pairs while refolding times were found to be slower, consistent with a general reduction in stem-loop stability caused by the AU/AT pair. Independent measurement of the dynamic timescales for melting and refolding of ring vibrational modes of guanine (G_R_) and adenine (A_R_) provided position-specific insight into hairpin dynamics. The G_R_-derived data showed that DNA sequences melted more quickly (0.5 ± 0.1 to 0.7 ± 0.1 μs at 70 °C) than analogous RNA sequences (4.3 ± 0.4 to 4.4 ± 0.3 μs at 70 °C). Position-sensitive data from the A_R_ modes suggests that DNA hairpins begin melting from the terminal end of the stem toward the loop while RNA sequences begin melting from the loop. Refolding timescales for both RNA and DNA hairpins were found to be similar (250 ± 50 μs at 70 °C) except for RNA_end_ and DNA_loop_ which refolded much more slowly (746 ± 36 and 430 ± 31 μs, respectively), showing that the refolding pathway is significantly impaired by the placement of AU/AT pairs at different points in the stem. We conclude that conformational changes of analogous pairs of RNA and DNA tetraloops proceed by different mechanisms.

## Introduction

Ribonucleic acid (RNA) and deoxyribonucleic acid (DNA) play important roles in the storage and expression of genetic information, the underpinning mechanisms of which feature several dynamic intermolecular interactions involving both nucleic acids and proteins. An appreciation of the structural dynamics of RNA and DNA is thus essential for our understanding of the cellular machinery and has the potential to provide a means to develop new medical therapeutics and technologies.^1^ An important question when considering the nucleic acids is why does DNA favor the double-stranded configuration while RNA adopts complex three-dimensional structures based on folded single-stranded base sequences, given their similar molecular structures that differ only via an extra OH group in the 2′ position of the ribose unit and the replacement of thymine bases with uracil in RNA?

One of the critical functional structures formed by RNA is the tetraloop hairpin. These motifs consist of a series of four unpaired bases closed by a double-stranded stem and function principally as nucleation sites for folding or as a basis for biomolecular recognition.^2,3^ While the four bases in the loop are not paired, their sequences and structures strongly influence RNA thermodynamics. In particular, the UNCG-type tetraloops, where N represents any nucleotide, are a common feature in RNA and of particular interest due to their unusual stability, with melting points found to be as much as 20 °C higher than other hairpins.^2^

To understand the molecular factors contributing to both sequence stability and the folding mechanisms that lead to tetraloop structures, nonequilibrium temperature-jump/drop (T-jump/drop) infrared (IR) spectroscopy experiments have been applied.^4−14^ T-jump initiation induces a rapid (few ns) rise in temperature, and IR spectroscopy is used to probe the subsequent melting dynamics of the double-stranded stem structures, as has been widely applied to nucleic acid sequences.^4−14^ In the T-jump/drop method, the use of short-path-length sample cells causes the T-jump to cool on timescales of ∼140 μs, more quickly than the tetraloop structures refold, providing the additional ability to probe refolding dynamics as the sample recovers to equilibrium.^7^

Application of T-jump/drop IR experiments to a 12-nucleotide T/UACG tetraloop with a four GC base-paired stem showed that the lifetime of melting of the RNA sequence was an order of magnitude slower than DNA, but that refolding occurred on a similar timescale.^7^ The differences in melting times were ascribed to the DNA structure adopting B-form helices, whereas, as a consequence of differing backbone structure and interbase stacking distance, RNA assumes the A-form, leading to a more stable stem structure for the RNA hairpin.^15^ The shared refolding rate demonstrated that, despite their molecular differences, RNA and DNA have similar rate-limiting steps for refolding: the rate of formation of base stacking in the stem sequence.^7,16−18^

**Figure 1:**
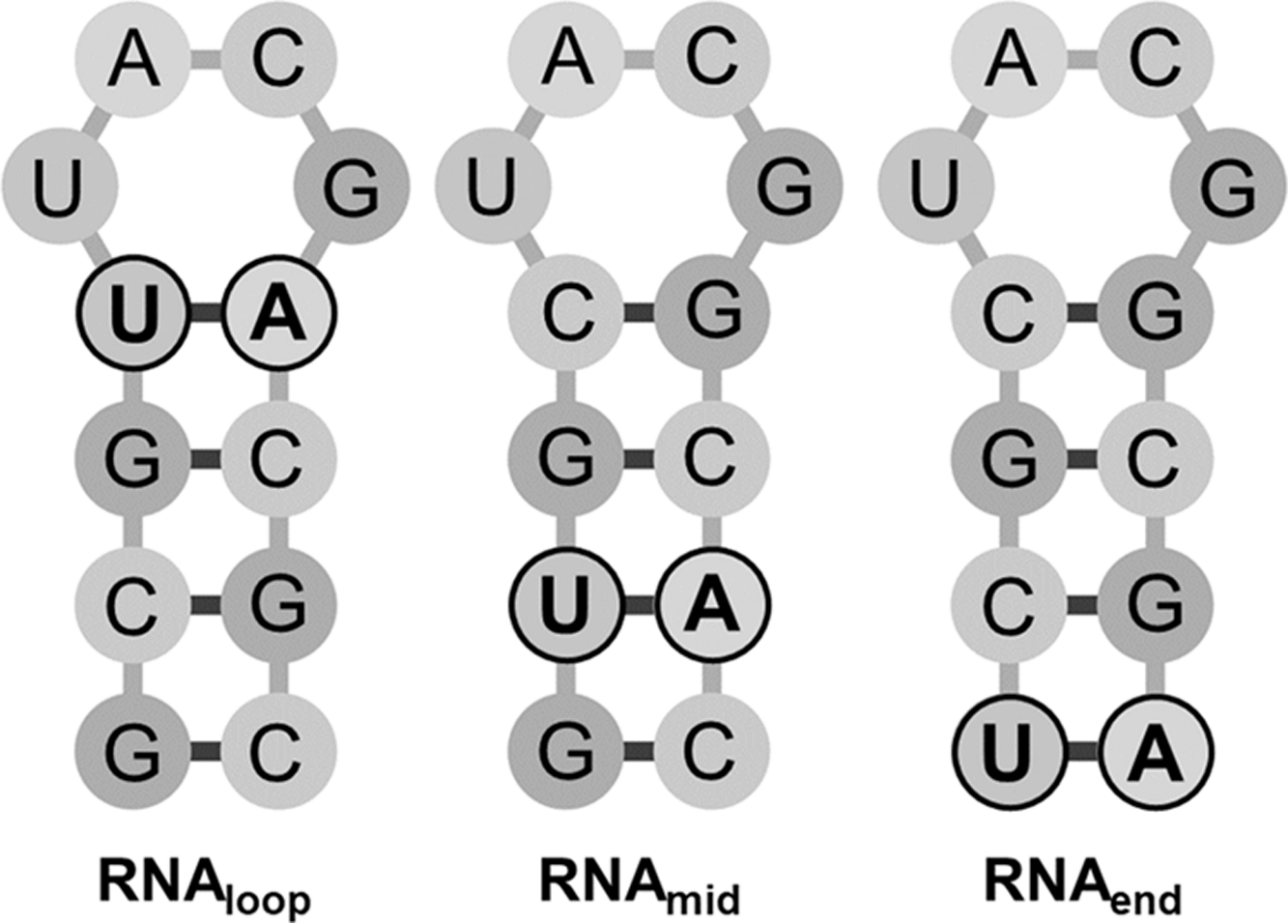
Schematic representation of the base sequences of the RNA UACG tetraloop hairpins (RNA_loop_ (left), RNA_mid_ (center), and RNA_end_ (right)) studied with T-jump/drop IR spectroscopy. The UA base pair position in the stem is highlighted in each case. The 5′ end is located at the bottom left of each structure.

The sequence of bases within the hairpin stems is likely to be a factor in determining their structure and dynamics. For example, it is known that, in nature, GC base pairs predominate at the closing position of the loop. To understand the details of the interactions in the hairpin stem and the mechanism of refolding, we extend our initial study of all-GC-containing stem structures with three stem sequences each featuring a single AU (RNA, Figure 1) or AT (DNA) inserted at specific points in the otherwise GC stem. The use of infrared spectroscopy to probe the response of the sequences following T-jump initiation allows clear differentiation of effects involving the main GC-rich stem and the single AU/T “label,” providing position-specific insight into changes in stem behavior arising from the AU/T inclusion.

The three label positions studied in RNA hairpins were the closing base pair (Figure 1, RNA_loop_), to determine the importance of the GC closing pair upon the rate of melting and refolding dynamics of the loop; the terminal end pair (Figure 1, RNA_end_) to compare the difference between the two ends of the stem and in the center of the stem adjacent to the terminal end (Figure 1, RNA_mid_). The data for the sequence with an all-GC stem (RNA_all_) characterized in previous work^7^ was used as a baseline comparison. In all cases, the DNA counterparts were also studied to extend our understanding of any dynamic differences between the two molecules.

Overall, we find that the general trends observed in the all-GC stem sample are repeated in the labeled samples. However, insertion of the AU/T pair at the closing point of the loop (DNA_loop_) and the terminal position (RNA_end_) has a significant impact on the refolding dynamics of the respective hairpins. From this, we conclude that RNA and DNA, while ostensibly similar in terms of refolding dynamics, refold via different mechanisms.

## Experimental Section

Salt-free, lyophilized RNA and DNA oligomer sequences, R/DNA_loop_: 5′-GCGX(XACG)ACGC-3′, R/DNA_mid_: 5′-GXGC(XACG)GCAC-3′ and R/DNA_end_: 5′-XCGC(XACG)GCGA-3′ (RNA: X = U; DNA X = T; “(···)” indicates the loop position) were purchased from Eurogentec. Deuterium oxide, potassium phosphate (mono and dibasic salts), and deuterium chloride were purchased from Sigma-Aldrich and used without modification. The base sequence of the GC component of the tetraloop stems was chosen to minimize base pair slippage.

The hairpin solutions all had a strand concentration of 10 mM, in 1 M deuterated phosphate buffer (pD 6.8), which is below the concentration limit where duplex formation occurs. All samples were annealed by heating to 95 °C for 5 min and leaving to cool for 1 h.^19,20^ For all experimental measurements, a 15–20 μL aliquot of nucleic acid solution was held between two, 2 mm thick, CaF_2_ windows mounted in a temperature-controlled cell (Harrick, ±1 °C).

Infrared absorption spectra were collected using a Bruker Vertex 70 Fourier transform (FT)-IR spectrometer. A sample path length of 50 μm, determined by a PTFE spacer, was used.

The temperature-jump spectrometer, using the STFC Central Laser Facility′s ULTRA B spectrometer, and the temperature-jump/drop method have been described previously.^4,7,21,22^ Briefly, the T-jump was initiated using a 4 ns duration pump pulse (125 Hz), generated by a Nd:YAG-pumped OPO, resonant with the high-frequency edge of the OD-stretching vibration of the D_2_O solvent at 2750 cm^–1^. This resulted in a T-jump of 10 °C, averaged across the sample, as confirmed using a calibration sample of trifluoroacetic acid (TFA).^4,21,22^ Probe pulses centered at 1650 cm^–1^ with a bandwidth of 300 cm^–1^ were generated by a Ti:sapphire-pumped OPA (10 kHz) with difference frequency mixing of signal and idler and were used to interrogate nanosecond to millisecond nucleic acid dynamics using the time-resolved multiple probe (TRMPS) approach.^4,7,21,22^ With this method, the molecular response was probed from 1 ns to 8 ms. It has been established previously that using a sample path length of 12 μm, defined by a PTFE spacer, establishes a T-jump cooling timescale, in the absence of R/DNA, of ∼140 μs (see below and ref (7)). In combination with the 4 ns rise time of the T-jump, this approach enables in excess of 80% of the expected amount of hairpin melting predicted by equilibrium IR absorption measurements to be observed, prior to full refolding.^7^ As the longest-measured relaxation timescale of the nucleic acid-containing samples to their starting condition was 746 μs (see the Results section), the final 4 ms of data were used as a “pump-off” measurement to obtain spectra that show differential absorbance.^4,21^

## Results

The sequence- and temperature-dependent melting and refolding behavior of the RNA UNCG tetraloop hairpins and their DNA equivalents were investigated using both equilibrium IR absorption and nonequilibrium T-jump/drop IR spectroscopy methods.

### Infrared Absorption Spectroscopy

#### Hairpin Melting

**Figure 2:**
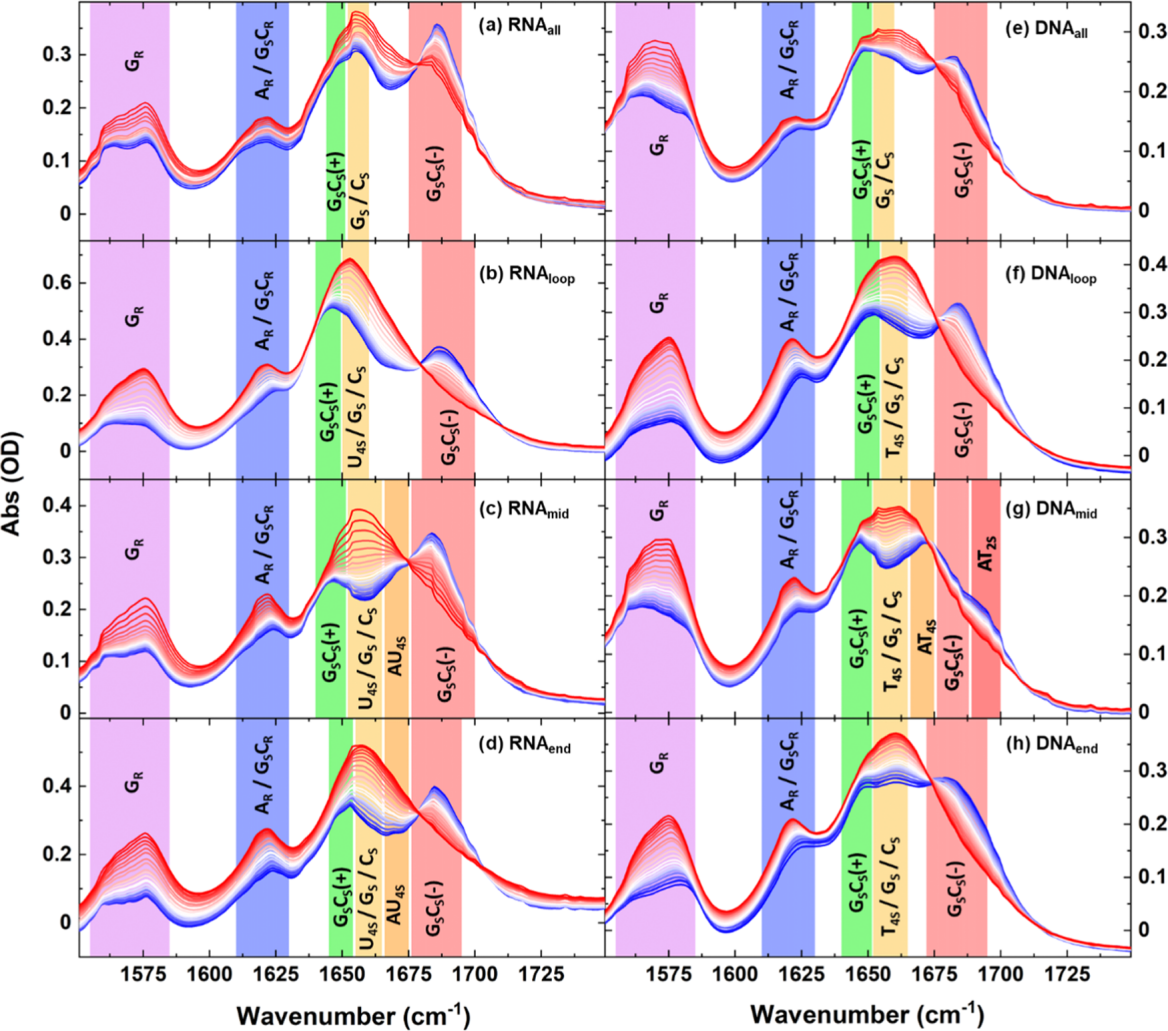
FT-IR spectra of RNA and DNA hairpins from 20 to 80 °C (blue to red) for RNA_all_, RNA_loop_, RNA_mid_, and RNA_end_ (a–d) and DNA_all_, DNA_loop_, DNA_mid_, and DNA_end_ (e–h). Vibrational mode assignments are indicated by colored panels: guanine ring mode, G_R_ (1575 cm^–1^, purple); overlapping adenine ring, A_R_ and base-paired guanine/cytosine ring mode, G_S_C_R_ (1620 cm^–1^, blue); symmetric base-paired guanine/cytosine mode, G_S_C_S_(+) (1648 cm^–1^, green); overlapping guanine, cytosine, and uracil/thymine stretching modes G_S_, C_S_, and U/T_4S_ (1656 cm^–1^, yellow); base-paired adenine-uracil/thymine stretching mode, U/AT_4S_ (orange, 1665 cm^–1^); base-paired asymmetric guanine-cytosine mode, G_S_C_S_(−) (1686 cm^–1^, red), and base-paired adenine and uracil/thymine stretching mode, AT_2S_ (1690 cm^–1^, dark red).^3,4,27^ The notation used to identify the vibrational modes of the bases is consistent with that used in refs (4) and (27). Spectra have been solvent corrected at each temperature. The data in (a) and (e) have been previously published in ref (7).

Infrared absorption spectra of the eight oligonucleotide hairpin sequences (RNA_all/loop/mid/end_ and DNA_all/loop/mid/end_) show a number of changes upon increasing the temperature from 20 to 80 °C (Figure 2a–h). The region of the mid-IR spectrum from 1550 to 1700 cm^–1^ (Figure 2) is dominated by vibrational modes of the nucleotide bases.^23^ Of particular relevance to this study of the melting dynamics of tetraloop hairpins with double-stranded stems principally composed of GC base pairs is the guanine ring vibrational mode (G_R_), which appears at 1575 cm^–1^ (Figure 2, purple panels). In all eight hairpins, the G_R_ mode was observed to undergo a significant increase in intensity between 20 °C (Figure 2, blue traces) and 80 °C (Figure 2, red traces). Plotting the intensity of the G_R_ mode as a function of temperature for each sequence (Figure S1) showed that, in all cases, the rise in intensity could be well described by fitting with a sigmoidal function. This behavior is consistent with hairpin melting, which refers to the dissociation of the base pairing and loss of base stacking in the double-stranded stems. This process is opposite to what occurs during double-stranded DNA formation whereby base stacking causes the extinction coefficient of the G_R_ mode to decrease, resulting in an increase in the intensity of the G_R_ mode upon melting.^4,5,7−9,23−27^ As the G_R_ mode also appears in a region of the nucleic acid spectrum that is relatively uncongested it has been used as a marker mode for GC base pair melting in nonequilibrium T-jump measurements below.^23,25^ The G_R_ band intensity was used to derive a melting curve for each hairpin, yielding the melting temperature (*T*_m_) and equilibrium thermodynamic parameters via the Van’t Hoff equation (Table 1). Being monomolecular and below the threshold for duplex formation, the derived thermodynamic parameters are concentration-independent.^19,20^ The *T*_m_ values are very similar for all six sequences with an AT or AU base pair included in the stem (loop, mid, and end), with all falling within the range 68 ± 5 °C. These values are all lower than the *T*_m_ exhibited by the R/DNA_all_ sequences, which yielded *T*_m_ values of 81 and 76 °C (±2 °C) respectively.^7^ The implication is that replacing a GC base pair with an AT or AU pair results in a reduction in the stability of the stem, irrespective of the position of the labeled pair.

**Table 1:** Dynamic and Thermodynamic Parameters Obtained from Analysis of IR Absorption and T-Jump/Drop IR Spectroscopy Experiments as Described in the Text

		RNA_loop_		RNA_mid_		RNA_end_		DNA_loop_		DNA_mid_		DNA_end_		
		G_R_	A_R_	G_R_	A_R_	G_R_	A_R_	G_R_	A_R_	G_R_	A_R_	G_R_	A_R_	
Van’t Hoff	*T*_m_	65	64	71	69	72	68	63	62	66	65	69	64	°C
	Δ*H*	203.9	184.8	154.4	140.5	148.6	137.2	154.8	178.0	148.4	165.9	123.9	188.8	kJ mol^–1^
	Δ*S*	603	548	449	410	431	403	46.1	531	437	491	362	557	J K^–1^mol^–1^
	Δ*G*^a^	17.0	15.0	15.4	13.3	14.9	12.4	12.0	13.3	12.9	13.6	11.7	16.1	kJ mol^–1^
	τ_1_^b^	4.3	4.3	4.3	4.5	4.4	7.6	0.7	2.0	0.7	1.4	0.5	0.4	μs
	τ_2_^b^	299		240		746		435		253		207		μs
Arr	*E*_a,m_	74.6	67.9	80.4	83.1	70.4	61.1	736.	25.2	58.9	32.2	74.2	54.4	kJ mol^–1^
	*E*_a,r_	–39		–54.9		–74.8		–43.2		–41.6		–24.0		kJ mol^–1^

a All Δ*G* values were calculated at 37 °C.

b Dynamic parameters (τ_1_ and τ_2_) are quoted at a temperature of 70 °C.

In the sequences RNA_loop/mid/end_ and their DNA equivalents, another band at 1625 cm^–1^ was observed to undergo a rise in intensity with increasing temperature (Figure 2, blue panels). This band is assigned to an adenine ring mode (A_R_)^23,25^ and the temperature dependence of the band intensity was also found to be sigmoidal in nature (Figure S2 and Table 1). The amplitude change is assigned to loss of base stacking/pairing of the single AU (RNA) and AT (DNA) base pairs placed in the stems of the R/DNA_loop/mid/end_ hairpins respectively in a similar manner to that of the G_R_ mode discussed above. As only a single base pair contributes to the intensity change of the A_R_ band upon stem melting, the increase in magnitude of the A_R_ band was found to be much smaller than that of the G_R_ band. Nevertheless, this mode serves as a proxy for the behavior of the AT/AU base pair in each hairpin and will be used as such in T-jump experiments below. It is however important to be aware of overlapping contributions from a number of different modes in this region that do not affect the G_R_ band.^23,25^

#### Sequence-Dependent Variations—RNA

The G_R_ and A_R_ modes allow assignment of the changes in the IR absorption spectra of the nucleic acid sequences as the temperature is increased to melting of the base-paired stem. By association, we assume concomitant loss of the hairpin structure. It is however also instructive to consider sequence-specific variations in the IR absorption spectra of the hairpins in the more complex spectral region between 1630 and 1700 cm^–1^.^23,25^

Considering the spectra of the RNA hairpins (RNA_all/loop/mid/end_) at 20 °C (Figure 2a–d, blue), it can be seen that two prominent bands near 1648 and 1686 cm^–1^ appear in all four spectra, which are attributable to the symmetric (G_S_C_S_(+)) and asymmetric (G_S_C_S_(−)) carbonyl stretching modes of the GC base pair, respectively (Figure 2, green and red panels).^23,25^ These bands arise due to the coupling of vibrational modes of the individual bases by Watson–Crick (W–C) H-bonding in the hairpin stem.^24,25^ As the temperature is increased, G_S_C_S_(+) appears to shift slightly to a higher wavenumber and increase in intensity as the strands melt while the G_S_C_S_(−) undergoes a large loss in intensity. More formally, the base-paired modes (G_S_C_S_(±)) are replaced by the uncoupled G_S_ and C_S_ modes of the unpaired bases (Figure 2, yellow), which results in a large, broad peak near 1660 cm^–1^ in the spectra of all four sequences obtained at 80 °C (Figure 2a–d, red traces).

Comparing the IR absorption spectra of all of the RNA hairpins at high temperatures (Figure S3a) shows that they are broadly similar, as would be expected when no secondary structure exists, though a small amount of the G_s_C_S_(−) mode is still visible in the RNA_all_ spectrum at 80 °C (Figure 2a), as a result of its slightly higher *T*_m_ value than the other hairpins. At lower temperatures (Figure 2, blue traces, and Figure S3a), other differences between the spectra of RNA_all_ and the RNA_loop/mid/end_ sequences become clear, which we infer arise from the inclusion of a single AU base pair at different points in the stems of RNA_loop/mid/end_.

In the case of RNA_mid_ (Figure 1) where the AU pair is located near the center of the GC-rich stem and so would be expected to cause minimal perturbation to the double-stranded structure, a shoulder is observed on the low-frequency side of the G_S_C_S_(−) band near 1675 cm^–1^ (Figures 2c and S3a, orange panel). We assign this band to the AU_4S_ stretching mode of the AU base pair, which is mainly due to the stretching vibration of the 4-position C=O bond in the W–C base-paired uracil base.^24,25,28^ The observation of the AU_4S_ mode is consistent with strong base pairing of the AU in the stem of RNA_mid_ as would be expected given its position. Upon stem melting (Figure 2c, red traces), the AU_4S_ band of RNA_mid_ is replaced by the U_4S_ mode of the unpaired uracil at 1660 cm^–1^ (Figure 2, yellow panel). This band overlaps with both the G_S_ and C_S_ modes, leading to a noticeably larger 1660 cm^–1^ band (Figure 2c, yellow panel) for RNA_mid_ in comparison to RNA_all_ (Figure 2a).

The spectrum of RNA_end_ at 20 °C (Figure 2d, blue) is similar to that of RNA_mid_ (Figure 2c). Although the AU_4S_ shoulder is not clearly visible, the G_S_C_S_(−) band is slightly broadened on the low-frequency side in comparison to that of RNA_all_. This suggests perhaps that in RNA_end_ the A and U modes are not as strongly coupled as in RNA_mid_, but that the AU is still in a base-paired configuration.

Conversely, the 20 °C spectrum of RNA_loop_ (Figure 2b, blue) is very different to those of RNA_all/mid/end_ with the relative intensities of the G_S_C_S_(±) bands reversed. Indeed, the G_S_C_S_(+) mode (Figure 2b, green panel) is both higher in intensity and broadened relative to those of RNA_all/mid/end_ and the general shape of the spectrum of RNA_loop_ at 20 °C is more similar to the spectra of RNA_mid/end_ at intermediate temperatures between 20 and 80 °C. We attribute this to a greater contribution to the 20 °C RNA_loop_ spectrum from the non-W–C U_4S_ mode. While such an observation could indicate some degree of fraying of the pair of bases at the closing point of the tetraloop, we note that a significant rise in intensity of the A_R_ mode (Figure 2, blue panel) with temperature is still observed for RNA_loop_. As the increase in A_R_ mode intensity is associated with the melting of double-stranded sequences, this suggests that the AU base stacking interactions are still in place. Thus, while there appears to be some disruption to the base pairing in the neck of the RNA_loop_ hairpin, which is not seen in the RNA_mid/end_ sequences, we do not attribute this to full base pair fraying.

#### Sequence-Dependent Variations—DNA

Applying a similar analysis to the IR absorption spectra of the four DNA sequences (Figure 2e–h) shows that, like their RNA counterparts, these too share very similar features at 80 °C although the slightly different position of T_4S_ relative to U_4S_ gives the peak near 1660 cm^–1^ a more symmetric profile in DNA than was observed in RNA (Figure 2, red spectra, Figure S3b, dashed lines). In the DNA hairpins, the G_S_C_S_(−) peaks are also generally less prominent than those of RNA.

Once again variations in the 20 °C spectra (Figure 2e–h, blue) provide insight into possible sequence-dependent differences in structural configurations caused by the positioning of the single AT base pair (Figure 2e–h, blue, Figure S3b, solid lines). At 20 °C, the spectrum of DNA_mid_ (Figure 2g, blue trace), with the AT pair in the center of the stem, contains contributions from the AT_4S_ (orange panel) and AT_2S_ (dark red panel) modes of the AT base pair. These bands occur at high and low frequencies relative to the G_S_C_S_(−) mode (red panel) and so significantly alter the lineshape and peak positions in the region from 1670 to 1700 cm^–1^. The presence of peaks deriving from the base-paired configuration of A and T indicates relatively strong AT base pairing in this sequence, consistent with the sharp rise of the A_R_ band alongside the G_R_ band (Figure 2g, blue and purple panels) with increasing temperature.

In the case of the DNA_loop_ hairpin, the G_S_C_S_(+) and G_S_C_S_(−) modes (Figure 2f, green and red panels) are of similar intensity to one another at 20 °C (blue). In this case, the AT_4S_ and AT_2S_ peaks observed in DNA_mid_ are not as clear, with AT_2S_ appearing as a weak high-frequency shoulder to G_S_C_S_(−), and there is a similarly weak contribution from the T_4S_ mode (Figure 2f, yellow panel) which is manifest as a broadening of the G_S_C_S_(+) band on its high-frequency side. Together, these observations are indicative of slightly reduced integrity of the base pairing near the neck of the tetraloop. The 20 °C spectra of DNA_all_ and DNA_loop_ are however very similar, suggesting minimal change due to the AT pair. As with the RNA_loop_ sequence, a rising intensity with temperature was observed for the A_R_ mode, consistent with the degree of disruption at the base of the loop being less than full fraying.

Finally, the 20 °C spectrum of DNA_end_ (Figure 2h, blue) shows that the G_S_C_S_(+) and G_S_C_S_(−) peaks form a plateau with the T_4S_ peak appearing distinctly between them (Figure 2h, yellow panel, Figure S3b, blue). This is a clear indicator of a weak terminal AT pair that is fraying to a significant degree, while the GC bases remain paired. Indeed, the absolute change in amplitude of the A_R_ mode of DNA_end_ between 20 and 80 °C was found to be less than half that of the other five sequences, consistent with a much-reduced loss of AT base pairing as the stem melted, as would be anticipated if the bases in the terminal position were already frayed. This is a notable difference between the RNA and DNA sequences, where no evidence of a frayed terminal AU was observed for RNA_end_.

#### Temperature-Jump/Drop IR Spectroscopy

Temperature-jump/drop IR spectroscopy was conducted on the RNA_loop/mid/end_ (Figure 1) sequences and their DNA analogues spanning a range of starting temperatures (*T*_0_) up to the point where the T-jump-induced temperature rise crossed their respective melting transitions (*T*_m_). This approach enabled insight into the influence of stem base sequence on the temperature-dependent melting and refolding dynamics of the hairpins.

The results of T-jump/drop experiments on the R/DNA_all_ sequences have been published previously^7^ where it was shown that the T-jump pulse heats the solvent quickly (ns), initiating melting of the hairpin stem, with RNA_all_ found to melt an order of magnitude more slowly than DNA_all_ (6 ± 0.1 μs versus 0.8 ± 0.1 μs at 70 °C). Solvent cooling was shown to occur on timescales of <150 μs, more quickly than hairpin refolding, allowing the refolding times of RNA_all_ and DNA_all_ to be measured, with both occurring on similar timescales of ∼200 μs at 70 °C.^7^

**Figure 3:**
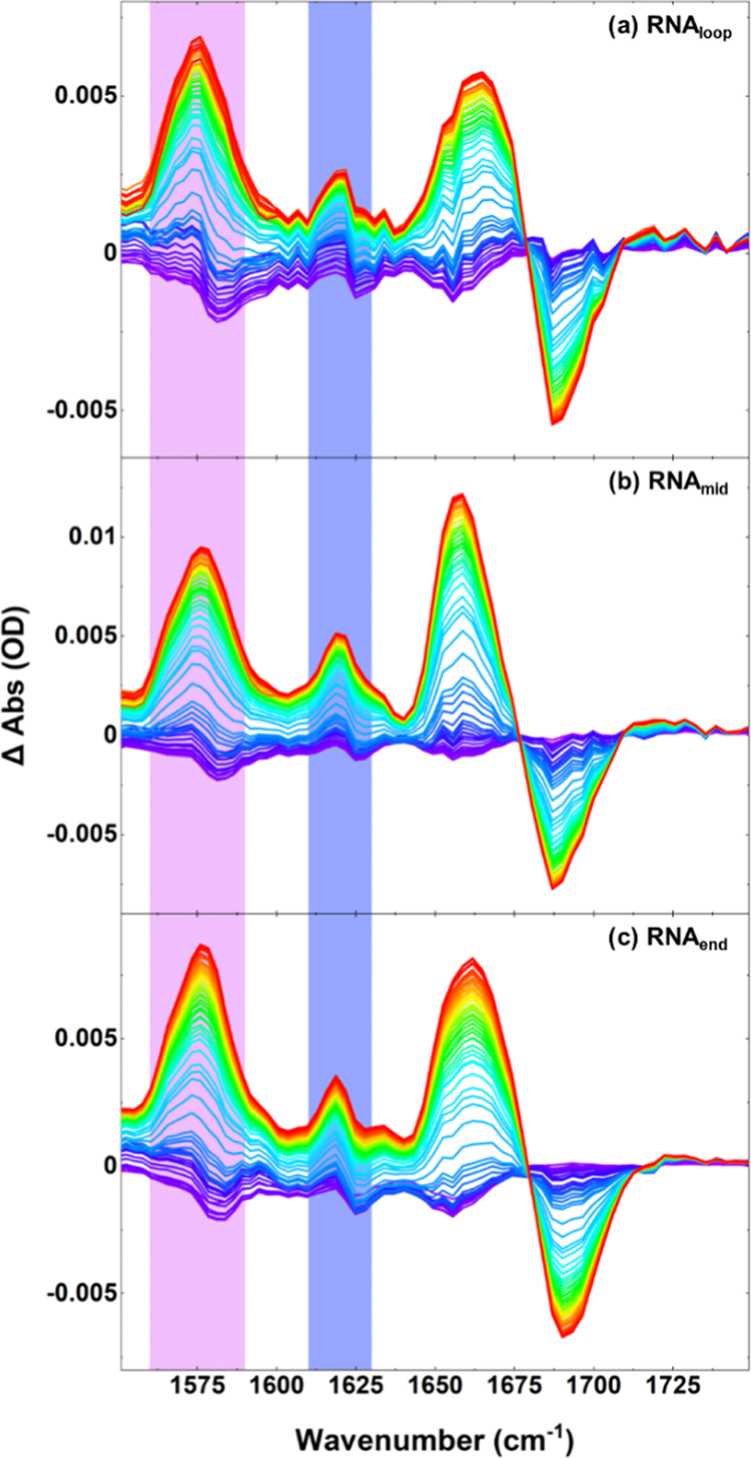
T-jump spectra for (a) RNA_loop_, (b) RNA_mid_, and (c) RNA_end_, showing the response of the hairpins from 1 ns (spectra colored blue) to the maximum signal obtained at T-jump-probe delay times of ∼10 to 20 μs (spectra colored red). Peak times vary by sequence. Spectra were obtained at *T*_0_ values of *T*_m_ – 5 °C. T-jump spectra are shown as pump-on minus pump-off difference spectra with the increase in amplitude of a band represented as a positive peak. The G_R_ mode at 1575 cm^–1^ and the A_R_ mode at 1620 cm^–1^, which are used as probes to convey the behavior of GC and AT base pair melting, respectively, have been highlighted in purple (G_R_) and blue (A_R_). The signals observed at very early T-jump-probe delay times in both datasets (blue traces) are due to fast hydrogen-bonding rearrangement.^7^ Spectra have been baseline corrected for visual clarity.

The T-jump spectra for RNA_loop/mid/end_, which all feature an AU base pair in the stem, are shown in Figure 3a–c for a starting temperature of *T*_m_ – 5 °C, with the equivalent data for the DNA sequences in Figure S4a–c. In all cases, the spectra are presented as pump-on minus pump-off absorbance difference spectra and show the changes in the spectra occurring over timescales from T-jump initiation to the sequence-dependent maximum signal, which was observed at T-jump-probe delay times of ∼10 to 20 μs (Figure 3).

For all RNA and DNA sequences, a sharp rise in intensity of the G_R_ (Figures 3 and S4, purple panels) and A_R_ bands (Figures 3 and S4, blue panels) was observed following the T-jump, establishing that stem melting is occurring. At longer T-jump-probe delay times, the peaks reduced in intensity toward the baseline. During this period, the spectral changes observed during the melting phase were reversed (Figures S5 and S6) and, by analogy with data for R/DNA_all_, we assign this to refolding of the hairpin stem as the sample cools following the T-jump.^7^

**Figure 4:**
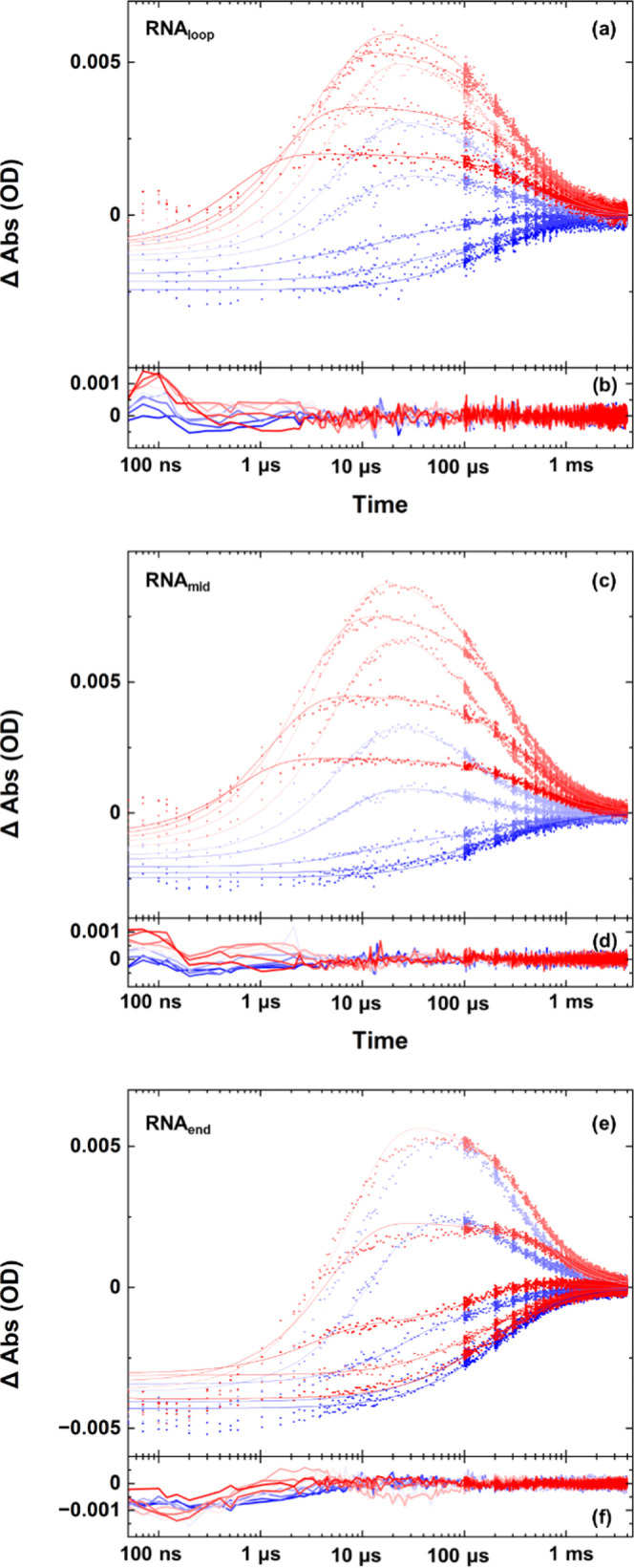
T-jump/drop dynamics showing temperature and time dependences of the G_R_ band of the RNA sequences (a) RNA_loop_, (c) RNA_mid_, and (e) RNA_end_. Data (dots) are shown from a *T*_0_ of 20–80 °C (blue-red) along with the results of fitting to a triple-exponential function (lines, see text). Residuals following the fitting process are shown in (b, d, f).

The melting and refolding dynamics for each of the R/DNA_loop/mid/end_ sequences were obtained by fitting the time-dependent amplitudes of the G_R_ and A_R_ modes to triple-exponential functions (Figures 4 and S7–S9). As established previously,^7^ the two major exponential time constants indicate the lifetimes of melting (τ_1_) and refolding (τ_2_). The third exponential term was generally of low amplitude (<25%) with lifetimes much longer than τ_2_. This was attributed to a slower dynamic process than refolding, but the small size of the signals made accurate determination challenging.^7^ It should be noted that D_2_O gives rise to a small, broadband solvent-dependent contribution to the data. This has not been subtracted from the data prior to fitting, but comparisons showed that applying this correction led to changes in lifetime parameters that fell within the errors stated.

#### G_R_ Melting Dynamics

**Figure 5:**
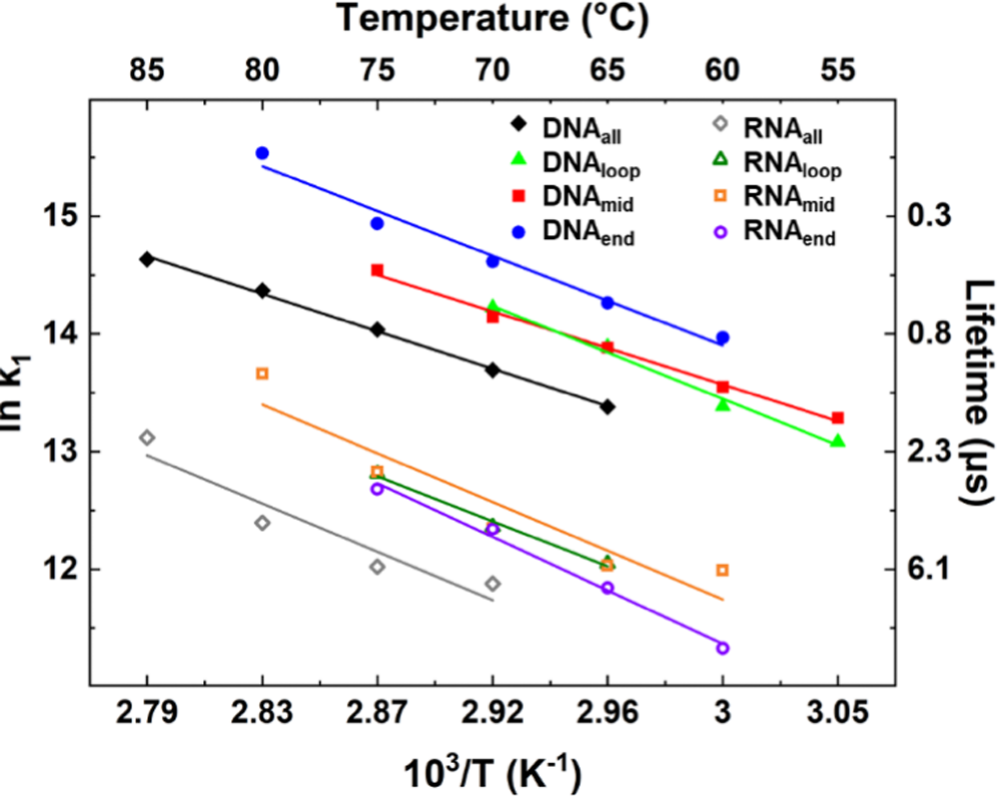
Arrhenius analysis of the temperature-dependent timescales of melting (τ_1_) determined via the G_R_ band for RNA (open symbols) and DNA (filled symbols) sequences. The data are shown over the temperature range where the maximum change in intensity of the G_R_ band was greater than 20% of the largest signal observed. Temperatures quoted are T_0_ + 5 °C, the average temperature over the T-jump.

The τ_1_ values obtained from fitting the time-dependent amplitudes of the G_R_ modes of the R/DNA_loop/mid/end_ sequences are shown in Figure 5, where it can be seen that all follow an Arrhenius temperature profile with a positive activation energy, consistent with previous observations.^7^ The D/RNA_all_ data are also provided for comparison purposes. Activation energies obtained from the Arrhenius analysis are shown in Table 1.

The lifetimes of melting with respect to the G_R_ mode represent an average over all of GC base pairs in the stem, though the link between G_R_ mode intensity and base stacking suggests that it will provide a good measure of the overall state of the hairpin stem at a given time after the T-jump.

Comparing the behavior of all sequences at a given temperature allows discussion of relative dynamic timescales free from Arrhenius-related effects on rates. Considering the RNA sequences, the melting dynamics determined for RNA_loop_, RNA_mid_, and RNA_end_ were consistent with one another, yielding lifetimes of 4.3 ± 0.4, 4.3 ± 0.3, and 4.4 ± 0.3 μs, respectively (Figure 5, dark green, orange, violet) at a T_0_ + 5 °C of 70 °C. As all sequences studied here show a similar *T*_m_, using a value of 70 °C probes a similar part of the melting curve for all sequences, as well as allowing comparisons with R/DNA_all_. A value of T_0_ + 5 °C is used to reflect the fact that the temperature jump was ∼10 °C on average across the sample. Henceforth, all temperatures quoted will be T_0_ + 5 °C unless stated.

The melting timescales of ∼4.3 μs for RNA_loop_, RNA_mid_, and RNA_end_ compare with a lifetime of melting of 6.9 ± 0.1 μs for RNA_all_ at the same temperature (Figure 5, gray). It is thus clear that inclusion of an AU base pair destabilizes the hairpin stems of RNA_loop/mid/end_ relative to that of the RNA_all_ sequence, while the similarities of the G_R_-derived melting timescales for RNA_loop/mid/end_ suggest that the proportion of AU to GC content exerts a greater influence on the melting dynamics than the specific sequence. This is also consistent with the similar reductions in *T*_m_ observed for RNA_loop/mid/end_ relative to RNA_all_ noted above.

For the DNA hairpins, the general pattern observed for the RNA samples of inclusion of an AT base pair leading to a shorter hairpin melting time was also observed, with DNA_loop_ and DNA_mid_ producing effectively identical τ_1_ values of 0.7 ± 0.1 μs, while DNA_end_ melted on a slightly shorter timescale at 0.5 ± 0.1 μs (at 70 °C). These compare with a value of 1.1 ± 0.1 μs for DNA_all_. Also noticeable is that the RNA sequences all melt with considerably longer timescales than their DNA counterparts, consistent with previous observations.^7^ The slightly shorter melting timescale for DNA_end_ relative to DNA_loop_ and DNA_mid_ could be associated with the observation of end fraying of the terminal base pair via IR absorption spectroscopy.

#### A_R_ Melting Dynamics

Unlike the information provided by the G_R_ band, the dynamic information derived from the A_R_ band provides site-specific insight, by virtue of there being only one paired adenine in each stem. By examining the dynamics of this band directly, a clearer understanding of how AU and AT inclusions at different points affect the stem can be established.

**Figure 6:**
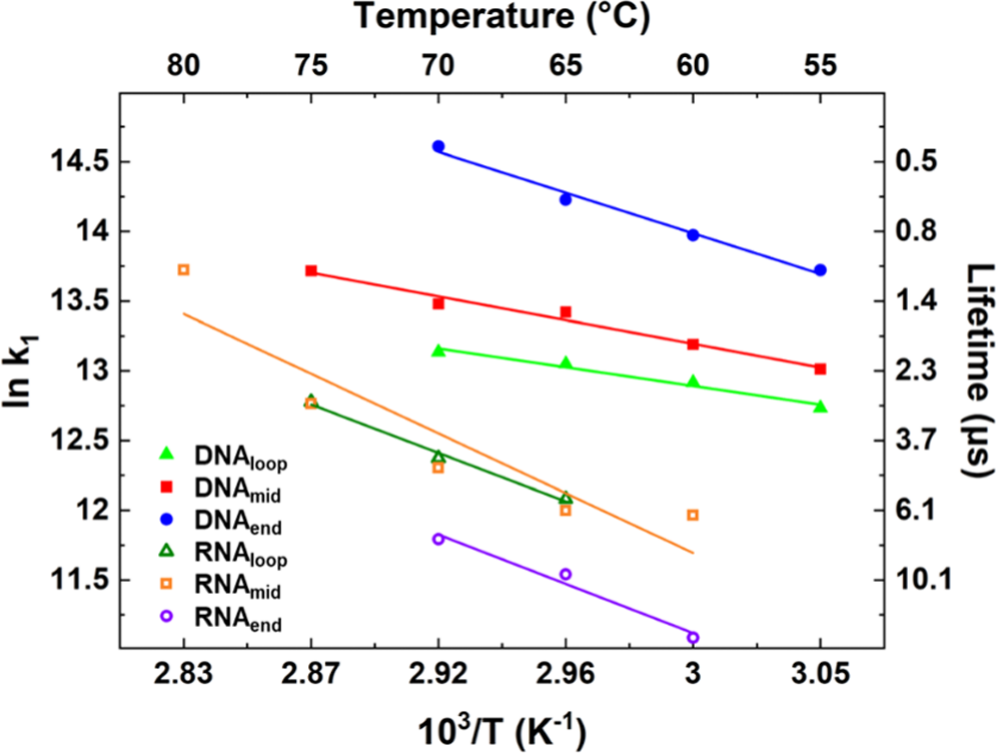
Arrhenius analysis of the temperature-dependent timescales of melting (τ_1_) derived from the A_R_ band of RNA (open symbols) and DNA (filled symbols) sequences. The data are shown over the temperature range where the maximum change in intensity of the A_R_ band was greater than 20% of the largest signal observed. Temperatures quoted are T_0_ + 5 °C, the average temperature over the T-jump.

The lifetimes of melting determined for the A_R_ modes (Figure 6) of RNA_loop_ and RNA_mid_, 4.3 ± 0.4 μs and 4.5 ± 0.1 μs (70 °C) were indistinguishable both from one another and from the τ_1_ values obtained for these sequences from the G_R_ mode. This indicates that the AU base pair is behaving as an integral component of the stem in these two cases. By contrast, the A_R_ melting timescale for RNA_end_ was found to be much longer, yielding a τ_1_ of 7.6 ± 2.0 μs at the same temperature. This implies that, rather than reflecting the dynamics of the main stem, the terminal base pair retains some degree of H-bonding or base stacking after the main part of the stem has dissociated, perhaps indicating the formation of a bubble-type structure prior to fully melting. Such a scenario would be consistent with IR absorption data suggesting that the terminal AU base pair is not prone to end fraying in RNA_end_.

In the case of the DNA hairpins, the A_R_ modes of DNA_loop_ and DNA_mid_ returned similar melting lifetimes of 2.0 ± 0.1 and 1.4 ± 0.3 μs, respectively, at 70 °C, slightly longer than the values obtained from the G_R_ mode for the same sequences. Conversely, the melting timescale of the A_R_ mode of DNA_end_ yielded a value of 0.4 ± 0.1 μs, shorter than the melting timescale for the G_R_ mode and consistent with DNA_end_ having a frayed terminal base pair that destabilizes the stem relative to the other sequences. It is noteworthy that this melting behavior, where DNA_end_ shows a faster melting time than the rest of the stem, is opposite to that seen for the RNA_end_ sequence.

#### Refolding Dynamics

**Figure 7:**
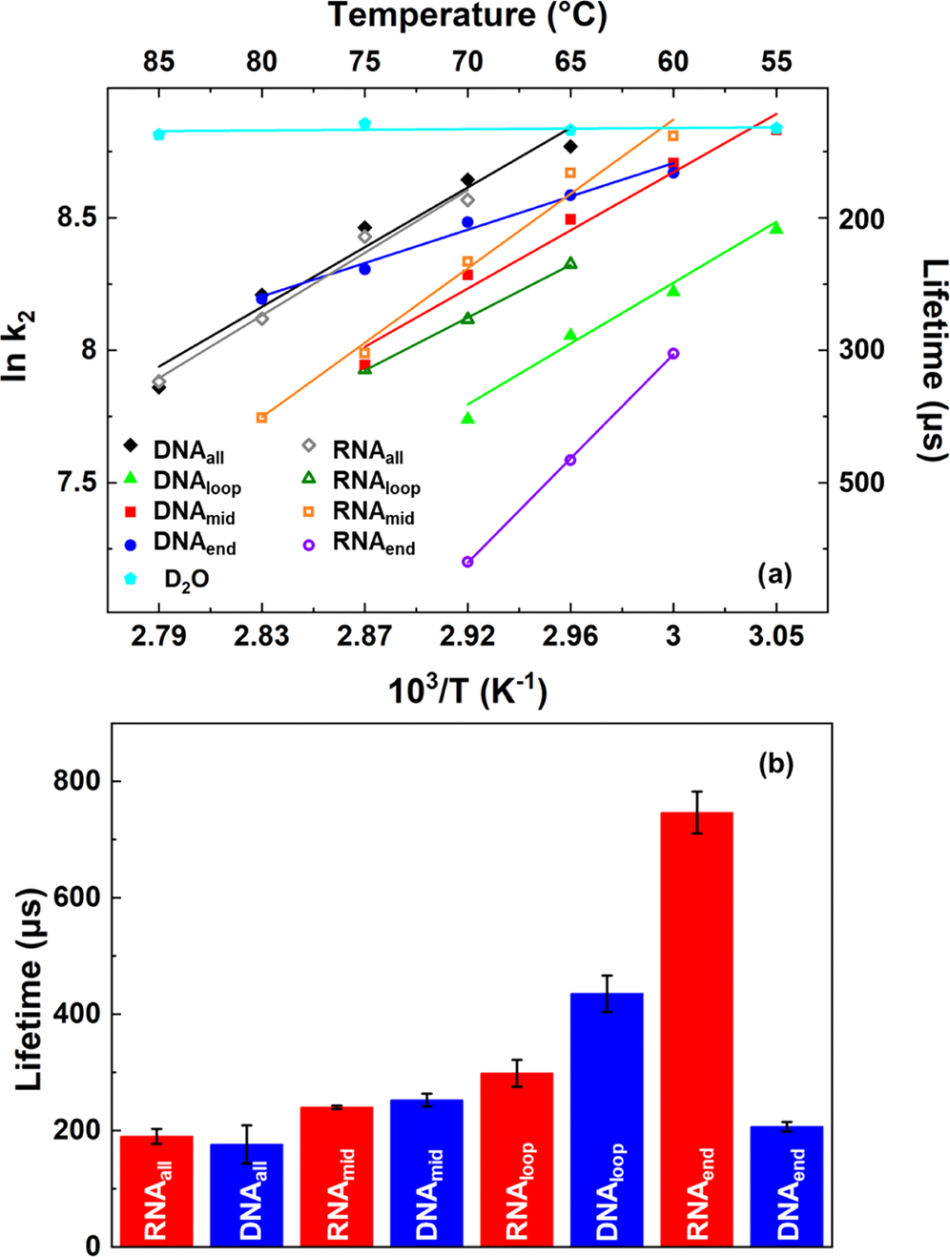
Temperature dependence of the lifetimes of refolding (τ_2_) derived from the G_R_ band of the RNA and DNA sequences. (a) Arrhenius analysis for RNA (open symbols) and DNA (filled symbols) sequences. The D_2_O solvent cooling dynamics, which are temperature-independent, are shown for comparison (cyan). Temperatures shown are T_0_ + 5 °C. (b) Bar graph showing a comparison of the lifetimes of refolding for all hairpins of RNA (red) and DNA (blue) at T_0_ + 5 °C of 70 °C.

It has been established previously that the cooling dynamics of the solvent following the T-jump are temperature-independent (Figure 7, light blue),^7^ and that the RNA_all_ and DNA_all_ hairpins were found to refold more slowly than the solvent cooling timescale of 140 μs. Furthermore, the hairpins were found to take longer to refold as the temperature was increased, which was manifest as a positive slope in the Arrhenius plot with apparent negative activation energies. These dynamics were assigned to a complex refolding landscape featuring a number of transient intermediate contacts between bases in the stem that did not lead directly to a fully base-paired and stacked stem.^7−10,12,29−33^ One of the consequences of this for T-drop spectroscopy is that at higher temperatures, the separation between refolding timescales and solvent cooling is greatest, minimizing the effects of convolution of cooling and refolding pathways.^7^

For the sequences with AT or AU base pair inclusions studied here, the refolding dynamics are reported based on only the G_R_ mode. This is due to the fact that the G_R_-derived signals were significantly larger than the A_R_ band, making fits of τ_2_ and τ_3_ more reliable.

Figure 7 shows the refolding timescales (τ_2_) obtained for all eight R/DNA sequences alongside the solvent cooling time (cyan). Anti-Arrhenius behavior is clear for all R/DNA sequences. Comparing the refolding dynamics (Figure 7b) of the individual sequences yields refolding times for RNA_mid_ and RNA_loop_ as 240 ± 30 and 299 ± 23 μs at 70 °C, respectively. By contrast, the value obtained for RNA_end_ was 746 ± 36 μs indicating that inserting an AU base pair at the terminal position significantly inhibits the refolding of the RNA hairpin. For the DNA hairpins, the trend was reversed; DNA_end_ and DNA_mid_ produced similar refolding timescales of 207 ± 8 and 253 ± 11 μs, while DNA_loop_ produced the longest τ_2_ value of 435 ± 31 μs (all at 70 °C). In this case, the AT base pair near the closing point of the loop appears to perturb the refolding process most dramatically.

In previous studies of the R/DNA_all_ sequences, it was found that although the melting rates were significantly longer for RNA compared to DNA their refolding timescales were very similar, with values of 190 ± 13 and 176 ± 33 μs being observed, respectively.^7^ Comparing these values to the data for the sequences with an AT or AU inclusion measured here shows that adding an AT/AU pair results in a generally longer refolding timescale. This suggests that refolding takes place more slowly, a fact consistent with both the reduction in *T*_m_ and the faster melting rates which accompany replacement of a GC base pair with an AT/AU pair, all of which indicate a less stable stem-loop structure. It is also notable that the refolding times of RNA_mid_, RNA_loop_, DNA_end_, and DNA_mid_ are all comparable, being on the order of ∼250 μs, as would be expected based on previous work.^7^ This leaves DNA_loop_ and RNA_end_ as clear outliers and shows that placing AU/AT pairs at these positions significantly inhibits the refolding pathway in each case.

It is interesting to compare the values obtained from the Arrhenius analysis with the trends derived directly from the dynamic timescales. Focusing on the G_R_-derived values shows that the activation energies for melting are all broadly similar. The values for refolding show a greater variation. In particular, the DNA_end_ and RNA_loop_ sequences (see Table) both show a small, negative activation energy that differs from the remainder of the sequences. It must however be noted that Arrhenius activation energy parameters are derived from the temperature dependence of the dynamic timescales and are enthalpic values only, thereby ignoring entropic effects, which could be substantial for mixed base sequences.

## Discussion

To understand the processes occurring and relate the similarities and differences in dynamics between analogous RNA and DNA hairpins to their structures it is important to draw on all of the evidence from the IR absorption spectra and T-jump/drop dynamics from the perspective of both the G_R_ and A_R_ bands.

### General Observations

It has been established previously, using tetraloops with all-GC stems, that RNA and DNA hairpins exhibit different melting timescales.^7^ This was attributed to differences in stem stability arising from the differences in base stacking between DNA and RNA, which can also be seen in the fact that they adopt differing helical structures, with RNA favoring the A-form and DNA the B-form.^34^ These conclusions also apply here based on T-jump data from the G_R_ mode, which acts as a reporter on overall stem dynamics. The RNA sequences were still found to melt more slowly than their DNA counterparts following site-specific inclusions of AU or AT base pairs into the stem. For both RNA and DNA, it was found that replacing a GC pair with an AU or AT led to a generally shorter melting timescale for the G_R_ mode, suggesting a relative destabilization of the stem-loop structure. This is in line with expectation based on the stronger pairing of GC bases compared to AU/AT because the former features three H-bonds, and the latter only two.

In terms of the refolding dynamics, all of the sequences with an AU/AT inclusion exhibited longer refolding timescales than the sequence with an all-GC stem (Table 1). The previous observation that RNA and DNA hairpins refolded on similar timescales^7^ was generally found to hold, but with two clear exceptions and we focus on these in more detail below. On the whole, the refolding process was found to be much slower than that of melting and can be attributed to the existence of more complex free energy profiles, with many more competing conformations and configurations on the potential energy surface to explore in comparison to strand melting. This mechanism is responsible for the anti-Arrhenius behavior and the apparent negative activation energies observed as, when considering the multiple possible intermediates, refolding is not a simple two-state process.^7−10,12,16−18,29−33^ Rather, we hypothesize that it is limited by the formation of the initial, correctly positioned, base pair and first stabilizing base stack.^16^

### RNA Tetraloops

Considering the G_R_-derived data, it is clear that the melting timescales of RNA_loop/mid/end_ are very similar and shorter than RNA_all_. When examining the behavior of the A_R_ modes for the individual structures, the site-specific labels of RNA_loop_ and RNA_mid_ track the G_R_ dynamics closely, enabling one to establish that they melt on comparable timescales. Such similar melting dynamics are consistent with a model where the conformation of the AU pair is governed by the rest of the GC stem. By contrast, the melting timescale of the A_R_ mode of RNA_end_ was found to be significantly slower than that of the other sequences, and slower than the GC melting behavior for the same strand.

From the IR spectroscopy data, the fact that the IR absorption spectrum of RNA_loop_ at 20 °C was similar in form to the spectra of RNA_mid/end_ at intermediate temperatures between 20 and 80 °C provided some evidence for a disrupted base pair at the closing point of the tetraloop, though the dynamic measurements suggest that this does not affect the melting timescales. Taking the evidence together, the implication is of an RNA hairpin that melts from the neck of the loop toward the end of the four base pair stem.

In terms of refolding dynamics, RNA_loop_ and RNA_mid_ were again found to behave similarly, with comparable melting timescales that were slightly longer than those of the RNA_all_ sequence, consistent with a reduced driving force for reassociation. Once again, RNA_end_ was an outlier with the refolding time of this sequence being most affected by base pair substitution and much longer than the other sequences. This shows that having an AU rather than GC at the end of the stem inhibits refolding. Given that this base pair was also found to exhibit a long melting lifetime, these two pieces of evidence could imply a significant degree of flexibility in the AU base pairing geometry whereby it can accommodate a significant perturbation of its structure without breaking the H-bonds, but that these perturbed structures, when formed as transient steps on the refolding pathway, do not lead to the reformation of the stem. Overall, the data are consistent with a mechanism for RNA melting that occurs from the loop but refolds from the terminal end.

### DNA Tetraloops

Considering the G_R_-derived data for the DNA sequences with an AT inclusion, we observe significant differences in the spectroscopy and dynamics of the sequences, and by extension the unfolding and folding mechanisms compared to RNA. For example, DNA_end_ melted on a shorter timescale than DNA_loop_ and DNA_mid_, which were comparable. Using site-specific data from the A_R_ mode emphasizes this trend, with the A_R_ mode melting on timescales of 2.0, 1.4, and 0.4 μs for DNA_loop_, DNA_mid_, and DNA_end_, respectively. Combining the time-resolved results with IR absorption spectroscopy data indicates that the terminal position is likely to be substantially end-frayed in DNA_end_ and implies that the stem of the DNA_end_ hairpin is essentially composed of just three formal base pairs. The consequence of this is a less stable structure that inevitably dissociates on a shorter timescale. Conversely, the central AT pair in DNA_mid_, which showed strong IR spectroscopy evidence for being in a W–C H-bonded state via prominent bands associated with base-paired AT vibrational modes, melts on a longer timescale than that in DNA_end_, as would be expected. There is however evidence that the AT pair in this mid position lowers the stability of its neighbors from the shorter melting time for the sequence relative to DNA_all_. The nearest neighbor (NN) method predicts the difference in enthalpy to be as much as 24.7 kJ mol^–1^ in dsDNA, though this is not recovered in our hairpin samples from either the melting point or the activation energy for this sequence.^35−38^

The loop closing AT in DNA_loop_ does not show evidence from the IR absorption data for fraying like that found when the label is placed at the stem terminal end. The melting data support this through observation of the longest melting timescale for the A_R_ mode of DNA_loop_. Although it will lower the stability of its neighboring GC, this base pair will be influenced through base stacking by a sequence of three GC pairs in the remainder of the stem. Overall, the data combine to suggest that the DNA hairpins unfold from the frayed terminal end, though it is hard to rule out whether this is a consequence of the fact that the terminal AT base pair is more unstable, effectively creating an initiation point for unfolding. It is however noteworthy that the analogous terminal AU pair in RNA_end_ shows no evidence of fraying.

Turning to the refolding dynamics shows that the DNA hairpin which refolds with the shortest timescale is DNA_end_, then DNA_mid_, and finally DNA_loop_. In the case of DNA_end_, the implication is that refolding is driven by the formation of the sequence of three GCs near the loop. At the other end of the stem, DNA_loop_ showed the longest refolding timescale by a significant degree, which would tend to suggest that the base pair at the base of the loop is important for nucleation of the refolding process, consistent with the shorter folding times for both DNA_mid_ and DNA_end_, which both feature GCs in the “loop” position. Taken together, the data point toward DNA hairpins which unfold from the terminal end, but refold from the loop.

### RNA–DNA Comparisons

It is interesting to note the differing impacts of site-specific AU/AT base pairs on the stems of the RNA and DNA hairpin loops.

The dynamic data is consistent with a model whereby the RNA stem melts from the loop, with the terminal AU pair showing a propensity to remain in a W–C paired state after the bulk of the stem has melted. When RNA refolds, it appears to do so from the terminal end, with the placement of an AU pair in the end position significantly impairing refolding. In contrast, a similar analysis leads to the reverse model for DNA in which melting begins from the frayed end base pair, while the base pair at the closing point of the loop is most influential in the refolding process, behaving as the anchor point for reversing change.

At room temperature, two sequences showed a departure from an archetypal stem-loop configuration, RNA_loop_ and DNA_end_, with both showing IR absorption spectroscopy evidence for structural disruption. This was most marked for the DNA_end_ sequence. Since, as discussed above, no fraying is observed in RNA_end_ (in contrast to DNA_end_) this shows that the RNA structure, through its closer base stacking and ordered backbone hydration is better able to stabilize the terminal AU pair.^34,39,40^

The A-form helical backbone structure that RNA adopts, by providing additional stability through closer base stacking and additional hydration of the backbone sugars, is likely to be responsible for the overall slower melting dynamics of RNA.^34,39,40^ The data suggests that the breakdown of base stacking is an important step in the melting of the double-stranded stem in both DNA and RNA.^16−18^ It is noteworthy that for R/DNA_mid_, the only sequence without three consecutive GC base pairs, melting is not significantly accelerated in RNA or DNA relative to R/DNA_end/loop_, suggesting that the collective nature of the base-paired stem overcomes any breaking of a sequence of GCs by a central AU/AT pair. The enthalpy penalty for replacing a GC with an AU in a GC sequence is ∼33 kJ mol^–1^ in RNA, as determined by the NN method, 5 kJ mol^-1^ more than the penalty for similarly substituting an AT in DNA, a difference that aligns with the closer (tighter) base stacking in RNA.^41^

From the perspective of refolding, placing the label in the center of the stem leads to similar refolding dynamics for R/DNA_mid_. This is consistent with previous studies of hairpins with all-GC stems and again shows that having a GC base pair on either side of the label prevents any significant perturbation of the stem behavior.^7^

It is interesting, given the consistency of the refolding dynamics of R/DNA_mid_ that the respective activation energies for refolding were found to be −54.9 ± 4.3 and −41.6 ± 4.5 kJ mol^–1^. The differences may reflect some impact of entropic contributions to refolding. The complexities of using activation energy parameters to predict refolding behavior are also shown by RNA_loop_ and DNA_loop_. While yielding similar activation energies (−39.0 ± 0.8 and −43.0 ± 1.9 kJ mol^–1^, respectively), these sequences showed dynamic differences, with DNA_loop_ refolding on longer timescales than RNA_loop_. This difference in timescale confirms that the rate of base stacking in DNA_loop_ is slowed appreciably and, based on structural differences, we tentatively ascribe the rate differences to the loop closing position being particularly important in the nucleation and zipping of the hairpin sequence in DNA. In RNA_loop_, the IR data shows that the closing pair is only weakly associated, and yet it still refolds much faster than the RNA_end_ sequence. While we cannot eliminate the possibility that faster folding of RNA_loop_ may be due to it adopting a different motif altogether, for this particular sequence at least, it implies that the loop closing position is less limiting in reforming the loop in RNA than DNA.

The relative behavior of RNA_end_ and DNA_end_ is the most divergent, defining the extremes of the refolding lifetimes of all of the sequences. The refolding lifetime of DNA_end_ is relatively short, being most similar to DNA_all_, and this supports the view that it behaves chiefly as a stem of three GCs since the AT is frayed at room temperature. This is clearly not the case for RNA_end_, where the AU terminal bases appear relatively strongly paired and remain so for an appreciable period during stem melting.

The fact that replacing the RNA_end_ terminal base pair with an AU leads to slower refolding compared not just to DNA_end_ but all of the other sequences indicates the different relative importance of the terminal position in refolding compared to DNA. Since the limiting step is believed to be the formation of the first base pair and stabilizing base stacking, this implies the RNA terminal position is important as a base-pair nucleation site in this sequence. In general, the probability of a stabilized base pair forming may be similar in RNA and DNA, but their different structures and flexibility may influence where that particular position is in the sequence; RNA appears to favor nucleation from the terminal end, while DNA prefers to refold from the loop closing position. The difference between their activation energies for refolding of −74.8 ± 0.4 and −24.0 ± 2.6 kJ mol^–1^ for RNA_end_ and DNA_end_, respectively, is also indicative of the relative importance of these positions on the stability of their respective structures; they have the highest and lowest refolding activation energies of the sequences.

From a biological mechanism perspective, it is important to consider whether the nature of the loop structures may also play a role in determining the observed folding mechanism. Apart from closer stacking, the RNA A-form helix also has a wider helical diameter and adopts a slightly different loop motif.^34,42^ The UNCG tetraloop in RNA forms a specialized “Z-turn”, formed by a trans-sugar Watson–Crick interaction between the first and fourth bases, facilitated by the C2′-endo puckering of the third residue and the third and fourth ribose rings are configured in a head-to-tail orientation resulting in an O4′−π stacking contact.^43^ This is a key stabilizing structure, but it is relatively rigid and, as such, may favor nucleation of the stem at a point distant from the loop, as we observe.

In DNA by comparison, the third residue of the loop is able to freely form either the C2′-endo or C3′-endo conformations.^44^ This may account for a greater flexibility in adjusting to a different geometry, allowing the stem to form from the base of the loop, but could also explain why the UNCG structure is less stabilizing in DNA in general. It is noteworthy that in nature 60% of RNA tetraloops are closed by CG pairs and 20% GC (80% by some combination of G and C).^3^ The free energy difference (ΔΔG_37_°) between the two is 7.8 kJ mol^–1^, while a UA closing pair is 11.7 kJ mol^–1^ adrift.^45^ Some studies have determined that the difference in stability and geometry between even CG and GC closing pairs can cause the formation of different loop motifs altogether^46^ supporting our observation that an AU pair imparts more flexibility into the stem structure in comparison to a GC or CG. Based on our observations, the dominant presence of GC or CG pairs at the closing point of naturally occurring loops would not appear to be due to folding timescales, as the refolding dynamics of RNA_loop/mid/end_ do not correlate with having a GC or AT in this key position.

## Conclusions

We have demonstrated that using temperature-jump/drop IR spectroscopy alongside IR absorption measurements allows the impact of stem base sequence upon melting and refolding dynamics of oligonucleotide hairpins to be measured. The melting timescales of the RNA and DNA hairpins highlight key differences between them. The DNA hairpins are less stable than analogous RNA sequences, with the insertion of an AT at the terminal end causing the DNA hairpin to dissociate on the shortest timescale and the longest melting timescale observed when the label was placed in the loop closing position. This is consistent with evidence for fraying of the terminal base pair and suggestive of a melting mechanism, which progresses toward the loop. For RNA, melting timescales were found to be largely insensitive to stem base sequence, however, placement of an AU at the terminal position led to a long melting timescale, reminiscent of bubble formation, which we ascribe to increased conformational flexibility of the AU base pair and indicated a melting direction that proceeds away from the loop.

Refolding dynamics showed that DNA and the equivalent RNA sequence generally refold on similar timescales; however, placement of the AU/AT label in the RNA_end_ and DNA_loop_ positions significantly impaired the refolding pathway, leading to longer refolding timescales. These results suggest that the folding pathway in RNA initiates at the terminal end and propagates toward the loop, while in the case of DNA, there is a preference toward zipping from the loop closing base pair. Taken together, the melting and refolding data for DNA and RNA sequences show that the mechanisms differ significantly, and we attribute this to stronger base stacking in RNA, which affects melting while greater flexibility of single-stranded DNA sequences gives access to different transient structures en route to refolding.

## Supporting Information Available

Additional spectroscopy data: FT-IR melting curves and normalized spectra, T-jump spectra for nucleic acid hairpins, and fitting of T-jump kinetics (PDF)
