# Supplementary material for: Sequence-Dependent Melting and Refolding Dynamics of RNA UNCG Tetraloops Using Temperature-Jump/Drop Infrared Spectroscopy
Source: J Phys Chem B. 2023 Feb 14;127(7):1586–97. doi: 10.1021/acs.jpcb.2c08709 (PMC9969394; doi:10.1021/acs.jpcb.2c08709)
Supplement: Supplementary file 1 — jp2c08709_si_001.pdf [file jp2c08709_si_001.pdf]

Sequence Dependent Melting and Refolding Dynamics of RNA UNCG Tetraloops Using Temperature-Jump/Drop Infrared Spectroscopy

C.P. Howe,[1] G.M. Greetham,[2] B. Procacci,[1] A.W. Parker[2] and N.T. Hunt[1]

1) Department of Chemistry and York Biomedical Research Institute, University of York, Heslington, York, YO10 5DD

2) STFC Central Laser Facility, Research Complex at Harwell, Rutherford Appleton Laboratory, Harwell Science and Innovation Campus, Didcot, Oxon, OX11 0QX

Supporting Information:

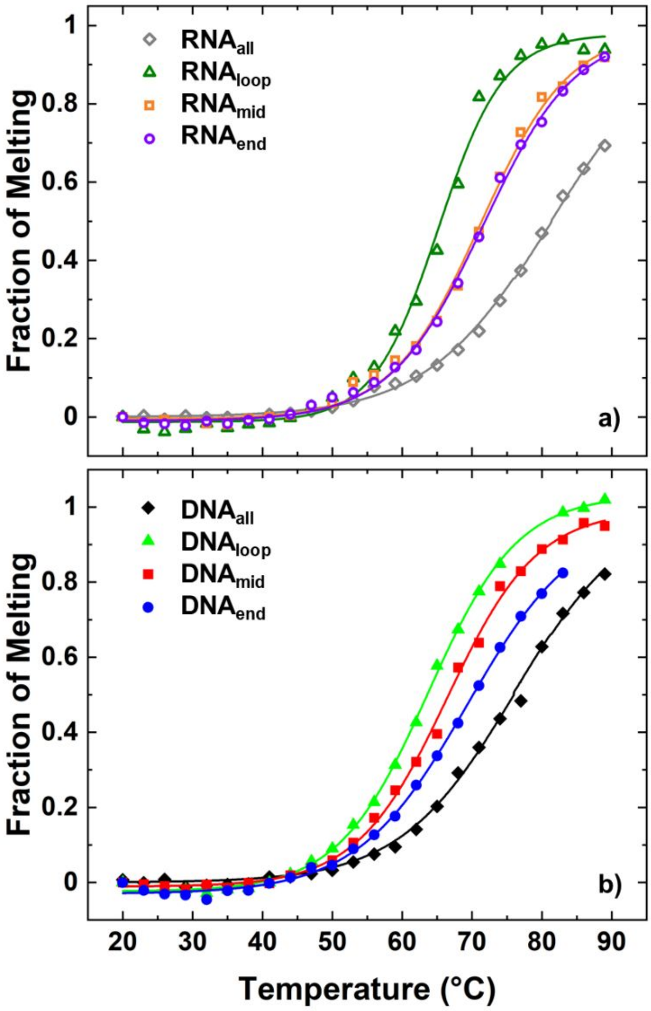

Figure S1. Melting curves for RNA sequences (a) and DNA sequences (b). These were derived from IR absorption spectroscopy experiments by plotting the intensity change of the G_R mode (1575 cm^{-1}) as a function of temperature. Lines show the results of fitting the data using sigmoidal functions. All datasets are normalised to their sigmoid fit maximum to show fraction of melting. All spectra were baseline corrected at 1750 cm^{-1}, which is in a region with no spectral features. A linear temperature-dependent contribution to the melting curves were extrapolated from the 20 – 40 °C range where no melting occurs and subtracted to give the data shown above.

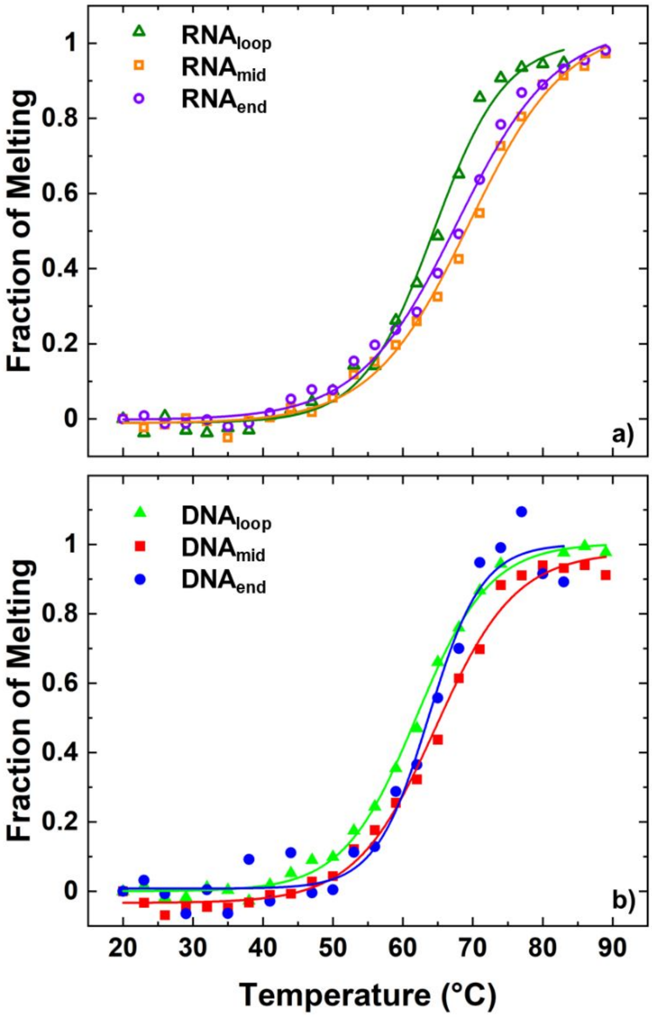

Figure S2. Melting curves for RNA sequences (a) and DNA sequences (b). These were derived from IR absorption spectroscopy experiments by plotting the intensity change of the A_R mode (1625 cm^{-1}) as a function of temperature. Lines show the results of fitting the data using sigmoidal functions. All datasets are normalised to their sigmoid fit maximum to show fraction of melting. All spectra were baseline corrected at 1750 cm^{-1}, which is in a region with no spectral features. A linear temperature-dependent contribution to the melting curves were extrapolated from the 20 – 40 °C range where no melting occurs and subtracted to give the data shown above.

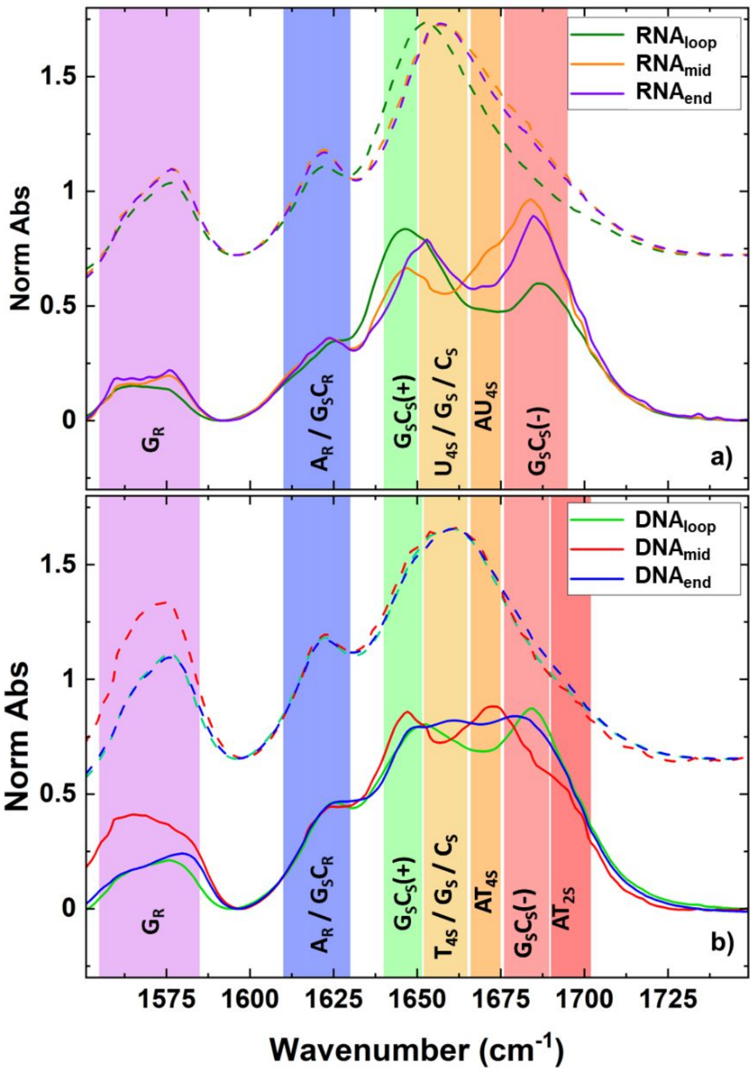

Figure S3: Normalised IR absorption spectra obtained at 20 °C (solid lines) and 80 °C (dashed lines) for a) RNA$_{loop}$ (dark green), RNA$_{mid}$ (orange), RNA$_{end}$ (violet) and b) DNA$_{loop}$ (green), DNA$_{mid}$ (red) and DNA$_{end}$ (blue). Prominent vibrational modes are indicated by colored panels (see main text). Spectra have been solvent and baseline corrected. All spectra are normalised to the 80 °C spectrum at 1656 cm^{-1} for the respective sequence.

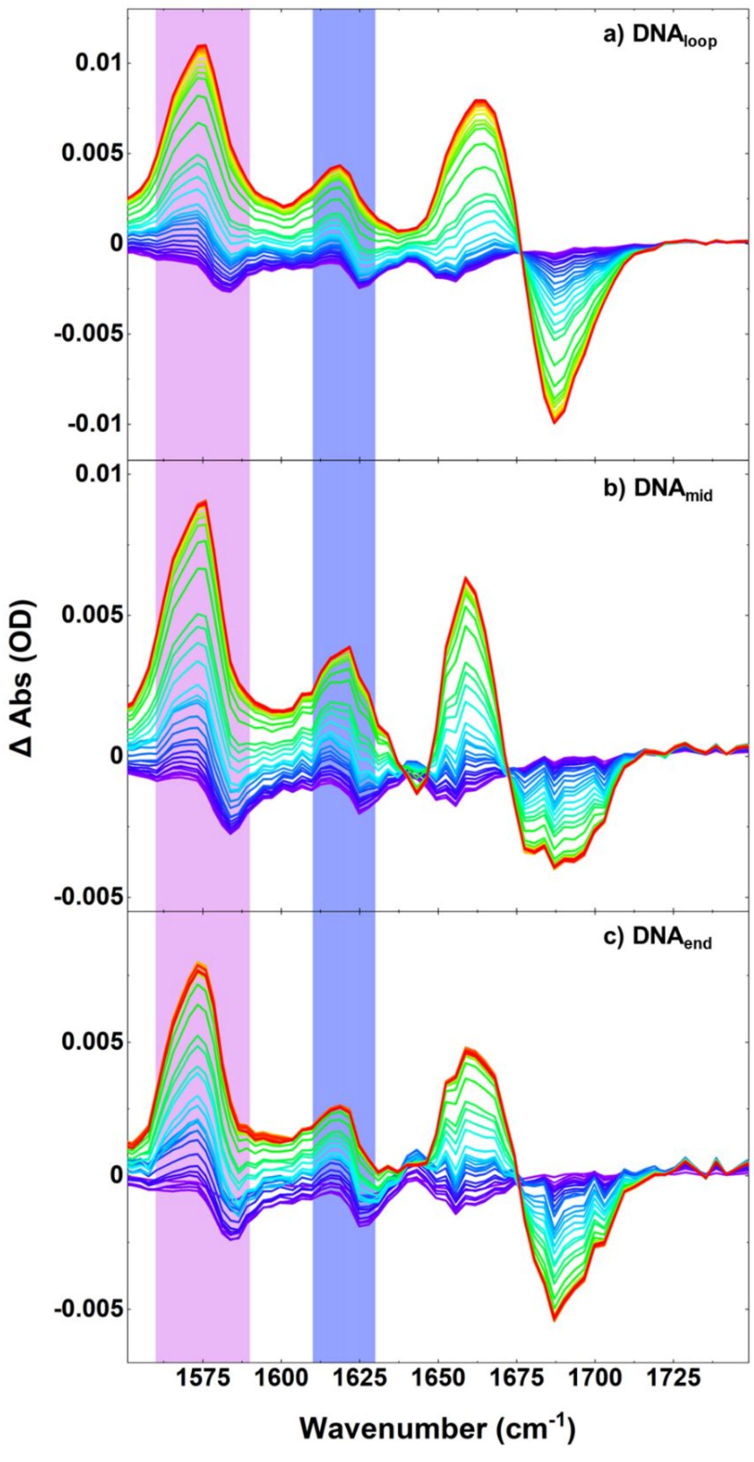

Figure S4: T-jump spectra for a) DNA$_{loop}$, b) DNA$_{mid}$, c) DNA$_{end}$, showing the response of the hairpins from 1 ns (blue) to maximum signal (red). Peak times vary by sequence. Spectra were taken at a T_0 nearest to T_m – 5 °C for each. T-jump spectra are shown as a pump on – pump off difference spectra with the increase in amplitude of a band represented as a positive peak. The G_R mode at 1575 cm^{-1} and the A_R mode at 1620 cm^{-1}, used as proxies for the behaviour of GC and AT base pair melting have been highlighted in purple and blue respectively. The signal seen at very early time is a fast hydrogen bonding rearrangement. Spectra were baseline corrected for visual clarity.

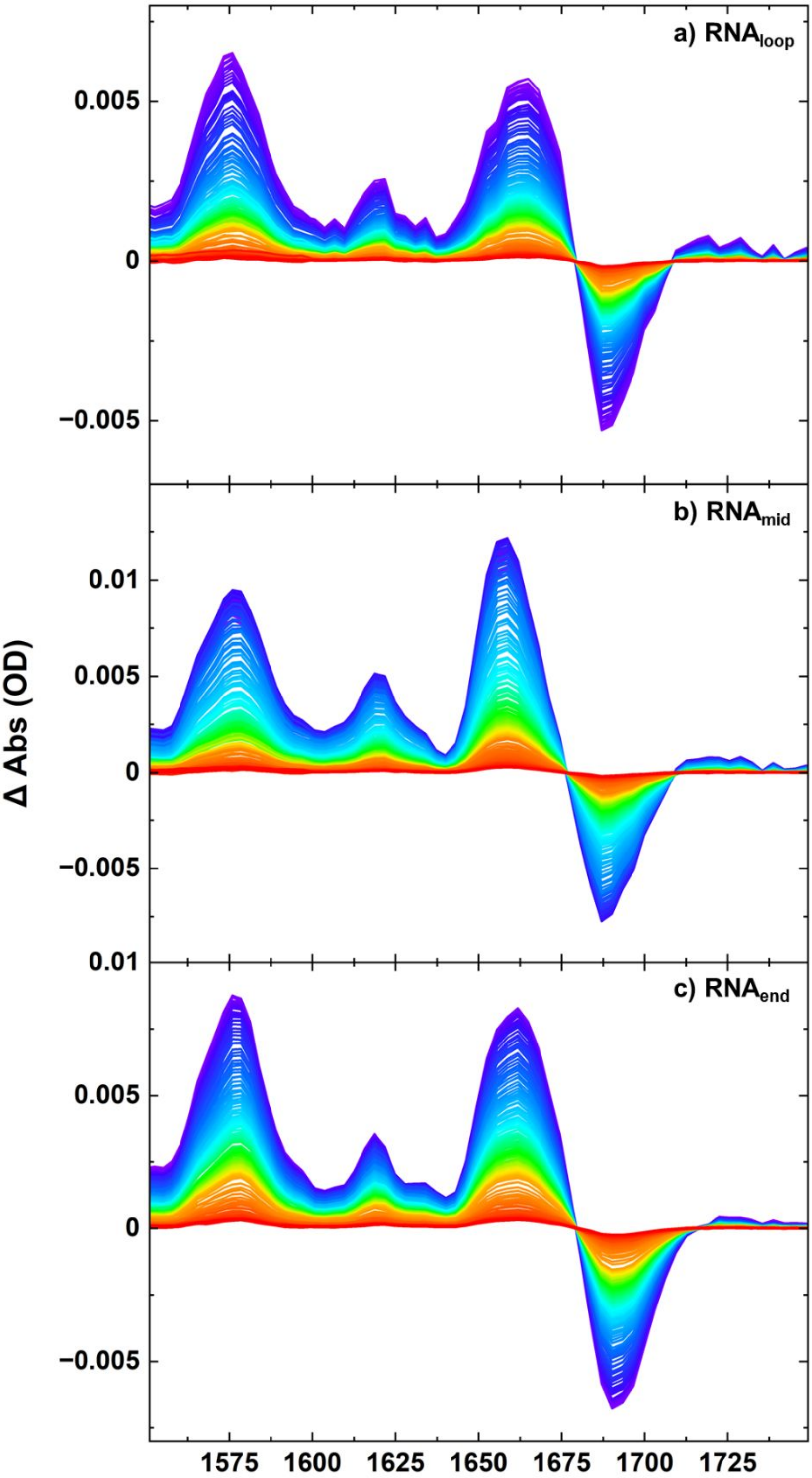

Figure S5: T-jump decay spectra for a) RNA_{loop}, b) RNA_{mid}, c) RNA_{end}, showing the decay of the hairpin signal from 24 µs (blue) to 4 ms (red). Spectra were taken at a T_0 nearest to T_m – 5 °C for each. T-jump spectra are shown as a pump on – pump off difference spectra with the increase in amplitude of a band represented as a positive peak. Spectra were baseline corrected for visual clarity.

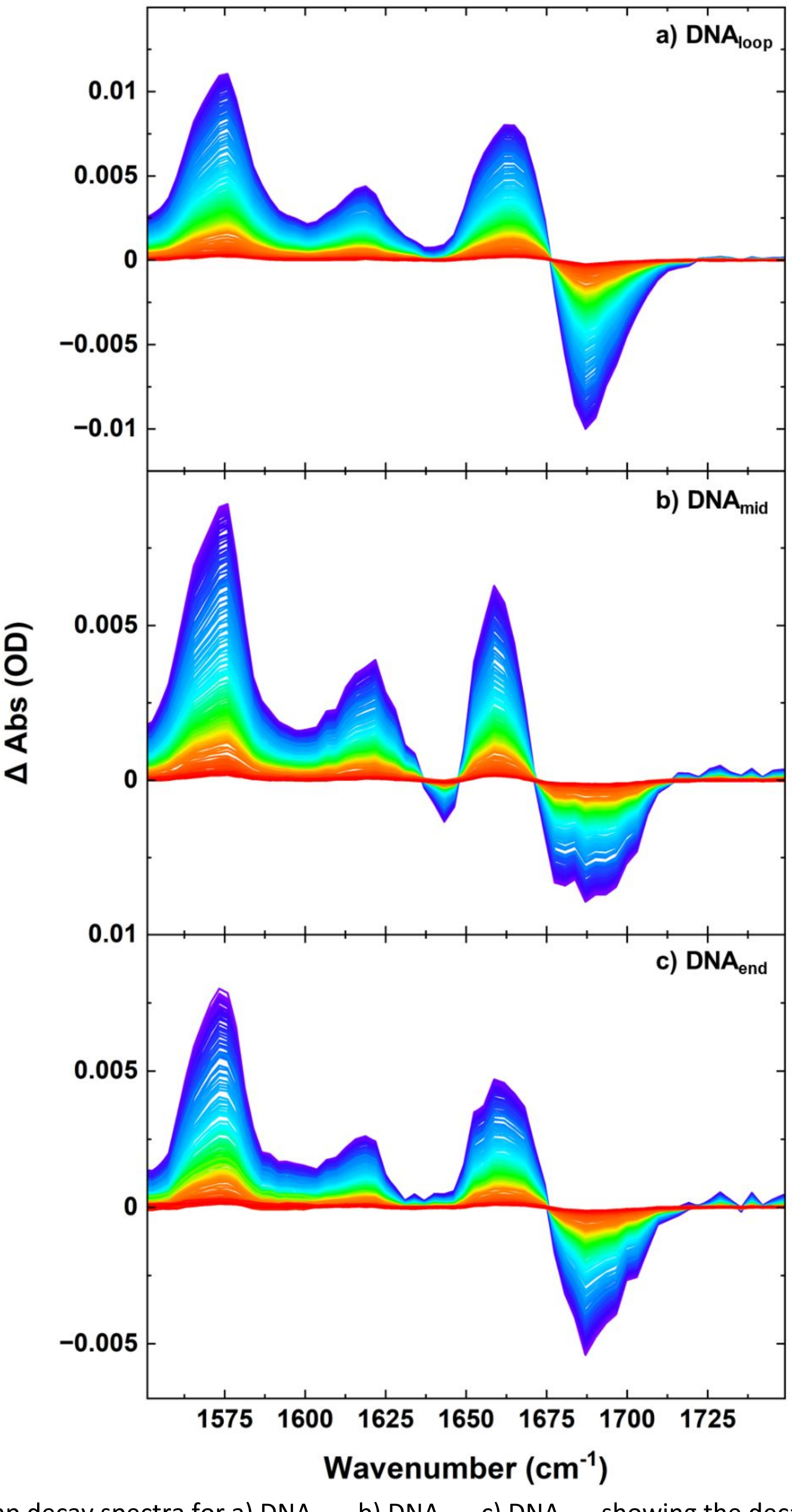

Figure S6: T-jump decay spectra for a) DNA$_{loop}$, b) DNA$_{mid}$, c) DNA$_{end}$, showing the decay of the hairpin signal from 6.8 µs (blue) to 4 ms (red). Spectra were taken at a T_0 nearest to T_m – 5 °C for each. T-jump spectra are shown as a pump on – pump off difference spectra with the increase in amplitude of a band represented as a positive peak. Spectra were baseline corrected for visual clarity.

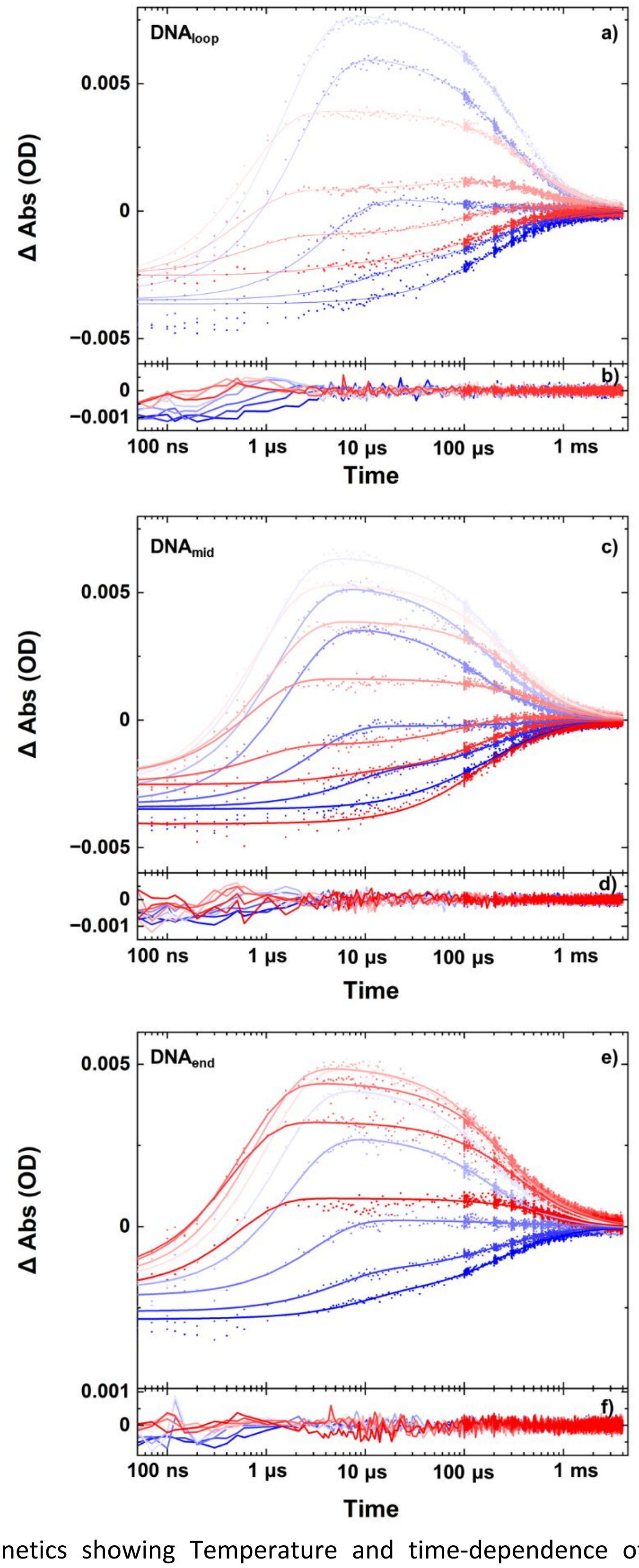

Figure S7. T-jump kinetics showing Temperature and time-dependence of the G_R of the DNA sequences a) DNA$_{loop}$, c) DNA$_{mid}$ and e) DNA$_{end}$. Data (dots) are shown from a T_0 of 20 °C to 80 °C (blue-red) along with the triple exponential fits (lines). Temperature dependent kinetic fit residuals are shown to scale below their respective kinetics (b, d, f).

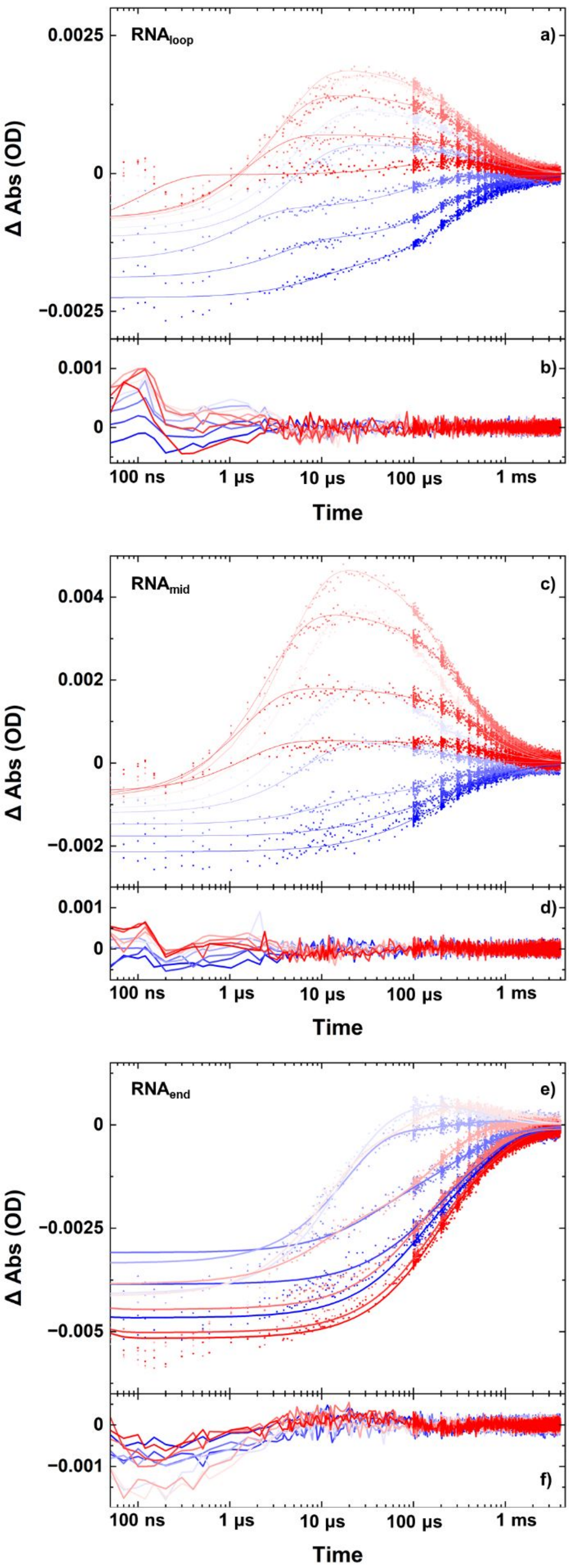

Figure S8. T-jump kinetics showing Temperature and time-dependence of the A_R of the RNA sequences a) RNA$_{loop}$, c) RNA$_{mid}$ and e) RNA$_{end}$. Data (dots) are shown from a T_0 of 20 °C to 80 °C (blue-red) along with the triple exponential fits (lines). Temperature dependent kinetic fit residuals are shown to scale below their respective kinetics (b, d, f).

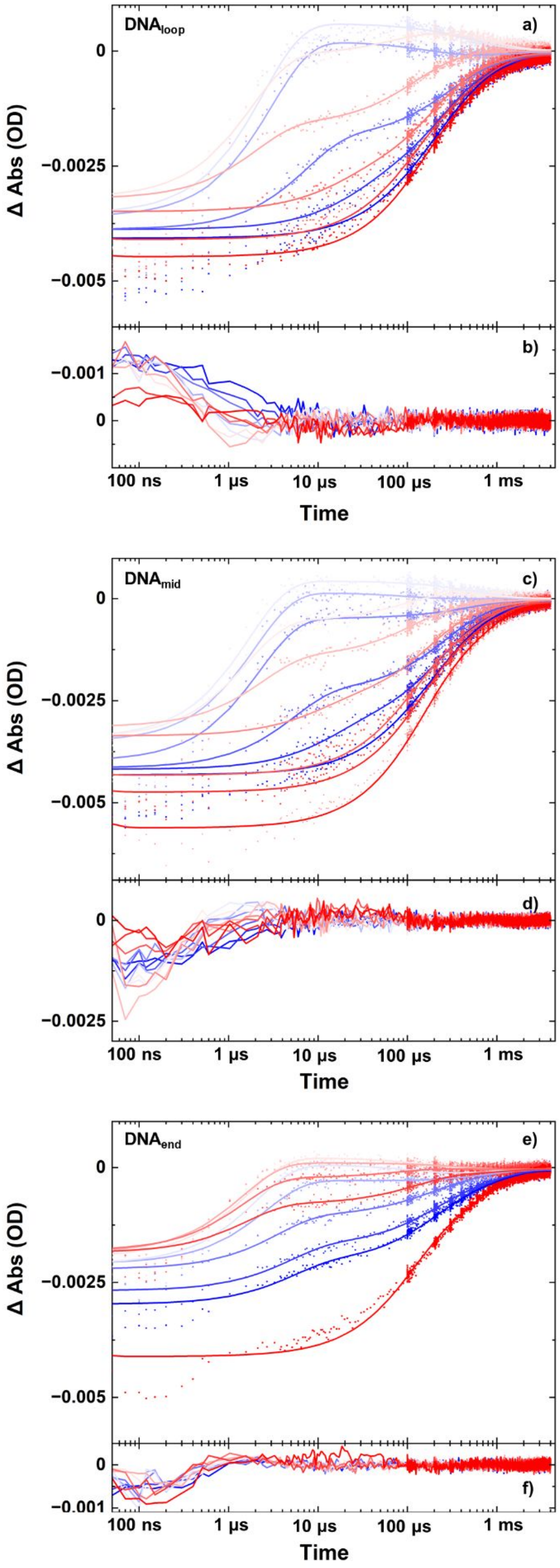

Figure S9. T-jump kinetics showing Temperature and time-dependence of the A_R of the DNA sequences a) DNA$_{loop}$, c) DNA$_{mid}$ and e) DNA$_{end}$. Data (dots) are shown from a T_0 of 20 °C to 80 °C (blue-red) along with the triple exponential fits (lines). Temperature dependent kinetic fit residuals are shown to scale below their respective kinetics (b, d, f).
